# Effect of an Additional 30 Minutes Spent Outdoors during Summer on Daily Steps and Individually Experienced Heat Index

**DOI:** 10.3390/ijerph17207558

**Published:** 2020-10-17

**Authors:** Suwei Wang, Molly B. Richardson, Connor Y.H. Wu, Benjamin F. Zaitchik, Julia M. Gohlke

**Affiliations:** 1 Translational Biology, Medicine, and Health (TBMH), Virginia Polytechnic Institute and State University, Blacksburg, VA 24061, USA; suwei@vt.edu; 2 Department of Population Health Sciences, Virginia Polytechnic Institute and State University, Blacksburg, VA 24061, USA; 3 Department of Medicine, University of Alabama at Birmingham, Birmingham, AL 35205, USA; mollyrichardson@uabmc.edu; 4 Department of Geospatial Informatics, Troy University, Troy, AL 36082, USA; yuhaowu@troy.edu; 5 Department of Earth and Planetary Sciences, Johns Hopkins University, Baltimore, MD 21218, USA; zaitchik@jhu.edu

**Keywords:** time spent outdoors, daily steps, summer temperature, wearable thermometer, physical activity

## Abstract

Spending time outdoors is associated with increased physical activity; however, high ambient temperature/humidity, together with built environment features in urban versus rural environments, may influence physical activity. We conducted an intervention trial with 89 urban and 88 rural participants performing normal activities on Days 1–2 (baseline) and spending an additional 30 min outdoors on Days 3–7 (intervention) in the summer. Participants wore a pedometer with real-time visual feedback to track daily steps taken and a thermometer clipped to their shoe to track temperatures experienced individually. Hygrometer–thermometers were deployed in participants’ neighborhoods to collect finer resolution ambient heat indexes in addition to regional weather station measurements. Using linear mixed effects models and adjusting for ambient conditions and individual-level factors, participants on average walked 637 (95%CI (83, 1192)) more steps and had a 0.59 °C (95%CI (0.30, 0.88)) lower daily mean individually experienced heat index during intervention days compared to baseline days. The intervention benefit of increased physical activity was greater in rural residents who were less active at baseline, compared to urban residents. Our results suggest adding a small amount of additional time outdoors may improve physical activity without increasing participants’ heat exposure, even during summer in a humid subtropical climate.

## 1. Introduction

Weather conditions, including high temperatures and precipitation levels, have been identified as barriers to participation in physical activity [1,2]. While physical activity generally peaks in summer [1,3], a lower level of physical activity in summer was noted in Texas residents when the average temperature of the study month was 29 °C (84 °F) [4]. Another study showed a moist tropical climate may be one of the strongest deterrents against physical activity in the U.S., reducing the percent of adults meeting physical activity recommendations by ~20% [5]. Adults in southeastern U.S. had the highest prevalence of physical inactivity (28.0%) based on Behavioral Risk Factor Surveillance System (BRFSS) data [6]. According to the World Health Organization, the prevalence of insufficient physical activity among adults is as high as 40–50% in countries in the subtropical or tropical zones such as Saudi Arabia, India, Brazil, etc. [7], where high temperatures may be a barrier to physical activity.

Different types of interventions (e.g., one-to-one counselling, self-directed physical activity, supervised physical activity, etc.) have been conducted to promote physical activity among children, adolescents, and older adults [8,9,10,11]. However, a review by Foster et al. (2014) suggested that the effect of interventions on self-reported physical activity was mixed with significant heterogeneity in reported effects [12]. New procedures are needed to meet perceived convenience, accessibility, safety, and aesthetic requirements in a given climatic condition, especially in humid tropical conditions [5].

As some studies have suggested, initiating and maintaining strenuous exercise programs is difficult [13,14]. Zimmerman et al. (2009) suggested the use of anchors such as social norms, habits and a cultural frame to influence people’s preferences for action to promote physical activity [15]. Nudging, which alters people’s behavior in a predictable way without forbidding any option, has been identified as an effective approach to promote physical activity [16,17]. For example, Bellettiere et al. (2017) found that stair use increased when placing signs at the bottom of stairs to encourage people to go up [18].

Evidence suggests that time spent outdoors is positively related to reduced sedentary time and moderate and strenuous exercise in adults [19,20,21,22,23]. Harada et al. showed that time spent outdoors was significantly and positively associated with physical activity measured as daily steps among 192 older adults, and suggested the health benefits of time spent outdoors were primarily mediated by physical activity [24]. Higher frequency of going outdoors was associated with less likely decline in the activity of daily living score among older adults [25]. Beyer et al. suggests the association between time spent outdoors and increased physical activity could be an opportunity to promote physical activity among youth [19]. However, the nudge approach related to time spent outdoors combined with technology support providing visible feedback (e.g., pedometer) for increased physical activity has largely been unexplored [17]. Encouraging even a small amount of additional time spent outdoors, which is positively associated with increased physical activity and reduced sedentary time from previous studies, could increase physical activity.

We hypothesized an intervention of spending an additional 30 min outdoors daily beyond normal activity would provide physical activity benefits with minimal risk to increased heat exposure, particularly when people are free to choose the time of day. Summers in Alabama (AL), U.S., are characterized by subtropical temperature and humidity, where the average high temperature is ~33 °C (91 °F), average low temperature is ~22 °C (71 °F), humidity is ~75% and there are ~12 days with precipitation per month [26]. Participants could freely choose time of the day and activity to spend an additional 30 min outdoors, and participants were instructed on methods to avoid heat stress and safely carry out the intervention. Effectiveness may be different across urban and rural settings; therefore, feasibility and compliance of this “nudge” intervention were estimated in both an urban and a rural setting in AL.

## 2. Materials and Methods

### 2.1. Participant Recruitment and Individual Level Measurements Collection

We screened and recruited urban residents of Birmingham, AL (N = 90) and rural residents of Wilcox County, AL (N = 90) in partnership with Friends of West End and West Central Alabama Community Health Improvement League during spring and summer 2017. Eligibility criteria included women aged 19–66 and availability to participate for seven consecutive days between 10 and 21 July 2017. We recruited women participants to reduce variability for the main outcomes of interest and to improve participant recruitment and follow-up based on our previous community-academic partnership research [27]. Additionally, women in AL are approximately 5% more likely than men to not engage in leisure-time physical activity [28]. Exclusion criteria included having medical conditions or taking medication that could prevent them from spending time outdoors or being out of town. Participants started participation on different days between 10 and 12 July 2017. Each participant completed seven consecutive days of participation, with study participation concluding between 17 and 19 July 2017. Potential participants attended an informational enrollment session, provided written consent, and filled out demographic questionnaires and a Physical Activity Neighborhood Environment Survey (PANES) [29,30]. We asked participants to perform normal activities on Days 1–2 and spend an additional 30 min outdoors beyond their normal activities on Days 3–7. We encouraged participants to choose time, activity, and locations to spend additional time outdoors to avoid dehydration, sunburn, or excessive exertion during the hot hours of the day. Participants kept a daily log of their outdoor time and pedometer readings. Participants received three phone calls to address any challenges in wearing the monitors and filling out the daily logs and they completed an exit survey. Data collection instruments are available at https://www.enactalabama.org/summer-2017. PANES score results are processed based on previously published methods, in which a valid score was assigned to participants who completed at least five out of the seven items [31].

Each participant was instructed to wear an iButton^®^ thermometer (DS1922L from Maxim Integrated, San Jose, CA, USA) clipped on the shoe and a pedometer (Yamax Digi-Walker SW-200 from Yamax, San Antonio, TX, USA) clipped at the waist in all waking hours, and leave them by their bedside during sleep. Participants clipped the thermometers facing down to avoid direct sunlight. Thermometers recorded temperature every five minutes. Participants were instructed to record their pedometer reading at night on each day without resetting. We collected the height and weight of participants with a stadiometer and a scale (Model PS660 from Befour Inc., Saukville, WI, USA) and body water, body fat, and muscle mass with a portable body composition scale (BC-553 from Tanita Corporation of America, Inc., Arlington Heights, IL, USA) at the beginning and end of participation. At turn-in sessions, we downloaded thermometer data and gave a printout of individual temperature results to participants. We stored all data on password-protected computers. This study was registered with www.clinicaltrials.gov (NCT 03614780) and approved by Virginia Tech Institutional Review Board (15-761).

### 2.2. Weather Station and Neighborhood Measurements Collection

We deployed 43 iButton thermometer–hygrometers (DS1923 from Maxim Integrated, San Jose, CA, USA) in participants’ neighborhoods. We placed each neighborhood thermometer–hygrometer in a radiation shield to avoid direct sun exposure [32]. We deployed the neighborhood thermometer–hygrometers at various locations (e.g., attached to trees in yards or along sidewalks) and recorded their latitude/longitude with a global positioning system (GPS). Neighborhood thermometer–hygrometers measured air temperature and relative humidity hourly. We accessed meteorological data, including air temperature, relative humidity, wind speed, precipitation, and location coordinates during the study from weather stations (WSs) in AL from the National Climate Data Center Surface Data, Hourly Global dataset (DS3505) [33].

### 2.3. Data Analysis

We geocoded participants’ home addresses using the World Geocoding Service in Arc GIS Pro desktop software (from Esri, Redlands, CA, USA). We matched each participant’s residence to the nearest neighborhood thermometer–hygrometer and the nearest WS. Six WSs in AL were matched to a participant home address (Supplemental File 1). We calculated the hourly WS heat index (i.e., HI[WS]) from WS temperature and relative humidity, and then calculated a daily mean and max HI[WS]. We calculated the hourly neighborhood heat index (i.e., HI[neighborhood]) from neighborhood averaged temperatures and relative humidity, and then calculated a daily mean and max HI[neighborhood].

A total of 178 participant thermometers (89 in Birmingham and 89 in Wilcox County) had valid temperature measurements at turn-in. We removed upper outliers of hourly averaged temperatures (646 out of 28,016 person-hours removed) which resulted in a dataset containing 27,470 person–hours of hourly averaged individually experienced HI (HI[individual]) (°C) from participant thermometers and matched WS relative humidity. We used “weathermetrics” packages for HI calculation in R [34]. We calculated daily mean and max of HI[individual]. The Intent-to-Treat (ITT) dataset contained HI[individual] of 1046 person-days. Based on daily logs, we removed 120 person-days of potential self-reported intervention-noncompliance (83 out of 522 person-days in rural participants, 27 out of 338 person-days in urban non-outdoor worker participants, and 10 out of 186 person-days in urban outdoor worker participants) to obtain a Per-Protocol (PP) dataset. We also performed analysis using the ITT dataset with no outlier removal.

An activity level was assigned to each participant based on reported weekly leisure activity levels in the Godin Leisure-Time Exercise Questionnaire [35]. We summarized self-reported intervention compliance (yes or no), reported difficulty in compliance, and reasons for non-compliance from daily logs. We explored factors associated with the probability of intervention compliance in a regression model accounting for ambient conditions and individual-level factors [36]. Unrealistic body composition values from five participants were removed (Supplemental File 1).

We calculated daily pedometer steps by:

Steps(N + 1) = Pedometer reading(N + 1) − Pedometer reading(N)(1)

where N ≥ 0 was day number. We removed person-days with negative steps as a minimally processed dataset. Building from our previous decision tree [37], we removed person-days with negative steps based on daily log notes and extreme daily steps <1000 or ≥25,000 [38]. We used this decision tree to further process steps as our primary pedometer dataset. We examined the differences between primary and minimally processed datasets in sensitivity analysis. Data collection and processing flowcharts are presented in Supplemental File 1.

We fitted linear mixed effects models to test whether steps changed, and whether participants daily mean or max individually experienced HI changed on intervention days compared to baseline, accounting for ambient conditions and other individual-level factors. Models included a random effect term to account for multiple measurements from each participant. We used “lmer” function from “lme4” package in R [39]. Primary analyses were Intent-to-Treat. Models include intervention, daily mean and max HI[WS] (°C), daily mean and max HI[neighborhood] (°C), WS daily mean wind speed (m/s), rain (yes or no), participant age, education (>high school vs. ≤high school), annual household income (>USD 20,000 vs. ≤USD 20,000), employment (yes or no), measured body fat (%), diabetic (yes or no), self-reported health condition (good, poor, fair), activity level (active vs. inactive), and an interaction term between intervention and groups. We determined whether to include HI[WS] or HI[neighborhood] or both to account for ambient conditions from model Akaike Information Criterion (AIC). We computed AIC for three models (both HI[WS] and HI[neighborhood], HI[neighborhood] only, HI[WS] only) and calculated the ∆_i_ = AIC_i_ − AIC_minimal_. The model best estimated has the ∆_i_ ≡ AIC_minimal_ ≡ 0 [40,41]. When ∆_i_ ≤ 2, there is no substantial difference between the two models [40,41]. If one model including HI[neighborhood] only and another model including HI[WS] only had ∆_i_ ≤ 2 with identical other fixed effects, the model including HI[neighborhood] only was reported because neighborhood thermometers were closer to participants’ homes than WS [42]. We ran models in separate groups to examine the intervention effect across urban and rural settings, and across occupationally and non-occupationally exposed groups. Measured body mass index (BMI) and measured body fat (%) were highly correlated, we included only measured body fat (%) in final models [27]. We dropped nine participants from the analysis because of missing measured body fat (%), annual household income, education, and self-reported health conditions. We ran sensitivity analysis models with intervention terms (intervention and weekday, intervention and weekend) to see if weekend changed the intervention effect. We performed additional sensitivity analysis described above using the ITT dataset with no outlier removal.

## 3. Results

Participants’ characteristics are presented in Table 1. We excluded one participant due to non-compliance with protocol (Consolidated Standards of Reporting Trials (CONSORT) flowchart in Supplemental File 1). All participants were women and 173 out of 177 (98%) participants self-identified as Black or African American. Thirty-two participants from Birmingham were outdoor workers (i.e., Urban OutWor). Urban OutWor participants were significantly younger (*p*-value 0.03), had a lower measured body fat (%) (*p*-value 0.04) and a higher measured body water (%) (*p*-value 0.02) compared to urban non-outdoor worker participants (i.e., Urban residents). Prevalence of diabetes was higher among Rural compared to Urban residents (35 out of 88 (40%) vs. 7 out of 57 (12%)). Rural participants on average had a higher measured body fat (%) (*p*-value 0.04) and a lower measured body water (%) (*p*-value 0.02) compared to Urban residents. We observed no significant differences in education, annual household income levels, BMI and obesity prevalence when comparing Rural vs. Urban residents, or Urban OutWor vs. Urban residents. When compared to the U.S. census data in these two locations, a higher percent of the participants self-identified as African American (95% vs. 71% in Birmingham, 100% vs. 71% in Wilcox County), had high school and above education (91% vs. 86% in Birmingham, 88% vs. 77% in Wilcox County), and had lower median annual household income (<USD 20,000 vs. USD 35,346 in Birmingham, <USD 20,000 vs. USD 27,237 in Wilcox County) [43]. A total of 166 out of 177 (94%) participants had a valid PANES score. Participants in the urban location had a significantly higher PANES score compared to participants in the rural location (3.4 out of 7 (95%CI (3.0, 3.7)) among participants in the urban location vs. 1.6 out of 7 (95%CI (1.3, 1.9)) among participants in the rural location).

**Figure 1:**
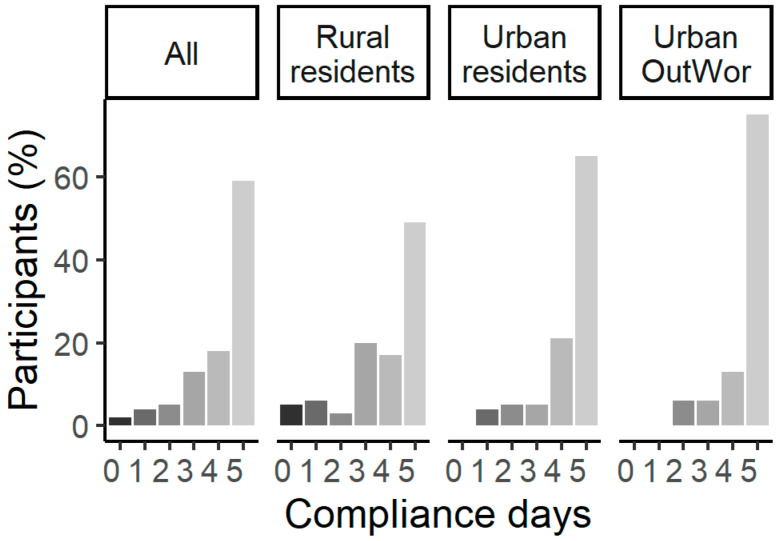
Intervention compliance days of participants. Participants (%) = number of participants/total participants × 100% in each compliance days category. Compliance days were days that participants carried out the intervention on Days 3–7 in the study. Pearson’s Chi-square tests showed the that there was significant difference in the distribution of compliance of person-days between Rural residents and Urban residents (Chi-Square = 7.99, Degrees of Freedom = 3, *p*-value = 0.046), but no significant difference between Urban residents and Urban OutWor (Chi-Square = 3.29, Degrees of Freedom = 3, *p*-value = 0.35).

Participants spent an additional 30 min outdoors on 736 (83%) intervention person-days. A total of 104 (59%) participants spent an additional 30 min outdoors on every intervention day while only four (2%) participants never carried out the intervention (Figure 1). There was a statistically significant difference in the compliance days between Rural residents and Urban residents (Chi-Square = 7.99, Degrees of Freedom = 3, *p*-value = 0.046), but no significant difference between Urban residents and Urban OutWor (Chi-Square = 3.29, Degrees of Freedom = 3, *p*-value = 0.35).

**Figure 2:**
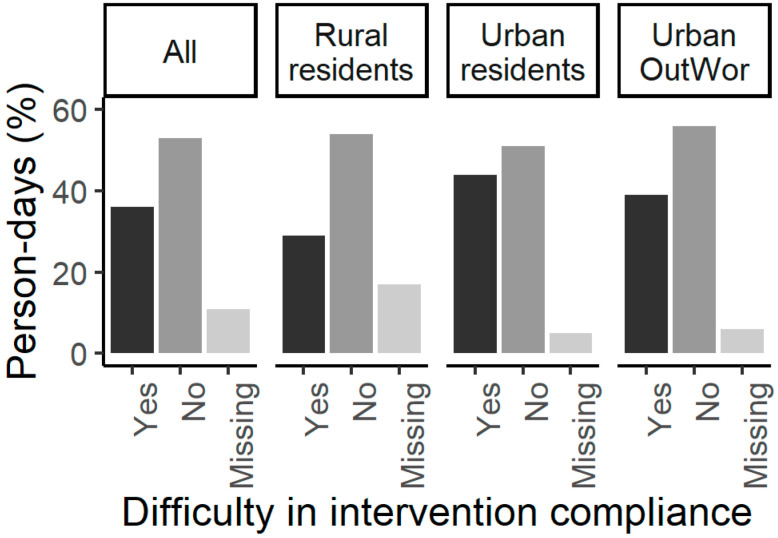
Person-days with self-reported compliance difficulty. Missing = participant did not report if she had difficulty in complying the intervention on a person-day. Pearson’s Chi-squared test showed that there was significant difference in the binary outcome of reported difficulty between Rural residents and Urban residents (Chi-Square = 8.83, Degrees of Freedom = 1, *p*-value = 0.003), and no significant difference between Urban residents and Urban OutWor (Chi-Square = 1.12, Degrees of Freedom = 1, *p*-value = 0.29).

**Figure 3:**
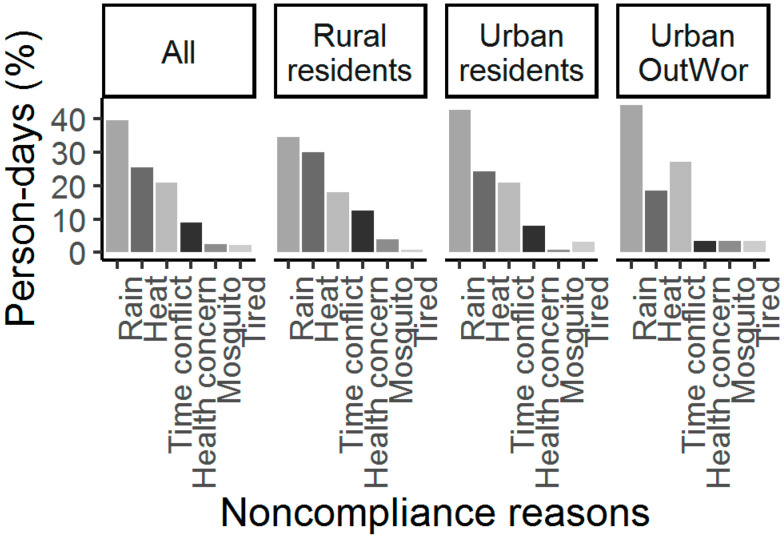
Self-reported noncompliance reasons of participants. There was no significant difference in the distribution of noncompliance reasons between Rural residents and Urban residents (Chi-Square = 3.04, Degrees of Freedom = 3, *p*-value = 0.39), or between Urban residents and Urban OutWor participants (Chi-Square = 2.93, Degrees of Freedom = 3, *p*-value = 0.39, *p*-value = 0.40).

The frequency of self-reported difficulty in intervention compliance is shown in Figure 2. Participants reported difficulty in intervention compliance on 316 (36%) person-days, and Urban residents reported more person-days with difficulty in intervention compliance compared to Rural residents (126 out of 285 (44%) person-days vs. 128 out of 440 (29%) person-days). We observed similar frequencies of reported difficulty between Urban residents and Urban OutWor (126 out of 285 (44%) person-days vs. 62 out of 160 (39%) person-days). The self-reported reasons for difficulty in intervention compliance are shown in Figure 3. Rain, heat, and time conflicts were the leading reasons for intervention compliance difficulties (Figure 3). We presented the factors associated with the probability of intervention compliance in Supplemental File 2. The effect sizes of most fixed effects are small; participants who were physically inactive had a 15.70% (95%CI (8.94%, 22.46%)) reduced probability of intervention compliance.

**Figure 4:**
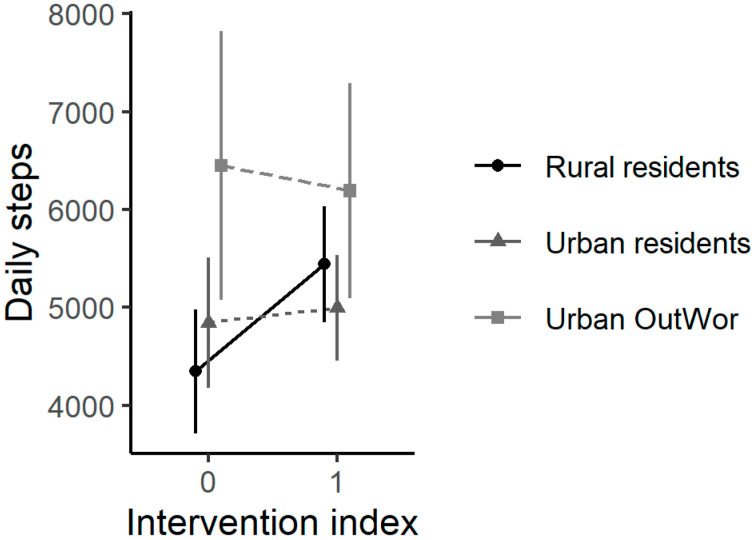
The population mean of individual mean daily steps on baseline days (intervention index = 0) and intervention days (intervention index = 1) in different population groups. The 95% confidence intervals are shown.

The population average of individual mean steps on baseline and intervention days is shown in Figure 4, where Rural residents and Urban residents walked more steps during intervention although the difference was statistically insignificant (Figure 4). In the mixed models, participants on average walked 637 (95%CI (83, 1192)) more steps on intervention days (Table 2). We did not find a significant interaction effect between intervention and groups (Supplemental File 3). In separate groups, Rural residents had a significant increase of 1063 (95%CI (273, 1851)) mean daily steps during intervention days, after accounting for ambient conditions and other individual-level factors (Table 2). Participants in urban locations had a smaller increase in steps on intervention days compared to Rural residents (Table 2). Participants walked more steps on intervention weekends than intervention weekdays (Supplemental File 4). Intent-to-Treat results and Per-Protocol results are similar; we found slightly lower estimated intervention effect in Per-Protocol, with an average 579 (95%CI (5, 1154)) additional steps on intervention days (Supplemental File 5). Participants had fewer steps on intervention days in the minimal processed dataset compared to primary dataset, with the β estimate of intervention −271 (95%CI (−960, 418)) in minimal processed dataset (Supplemental File 6).

**Figure 5:**
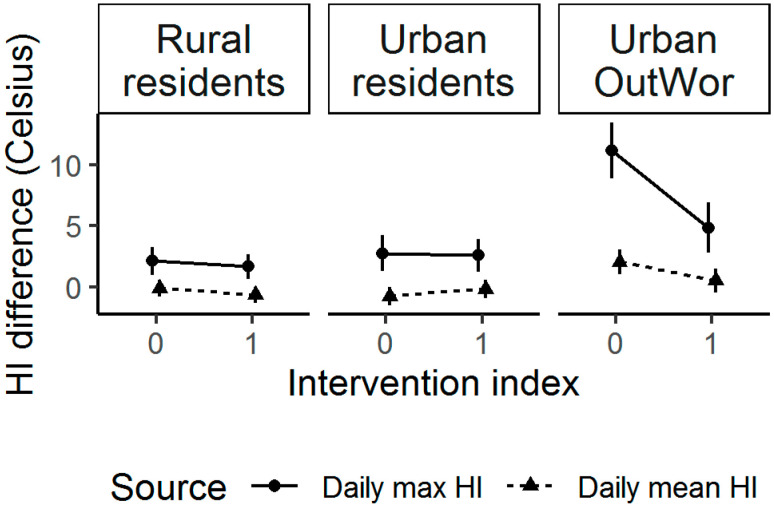
The mean HI difference (°C) between individually experienced HI and HI measured at the nearest weather station on baseline days (intervention index = 0) and intervention days (intervention index = 1). The 95% confidence intervals are shown.

Rural and Urban participants had similar average daily mean or max individually experienced HI on intervention days, but Urban OutWor had significantly lower daily mean or max individually experienced HI during the intervention, after accounting for WS HI (Figure 5). When we included ambient conditions and individual-level factors in models, we found overall participants had a 0.59 °C (95%CI (0.30, 0.88)) lower daily mean and a 1.40 °C (95%CI (0.53, 2.27)) lower daily max individually experienced HI on intervention days (Table 3 and Table 4). An interaction term between intervention and group was significant (Supplemental File 7). In separate groups, Rural residents and Urban OutWor participants on average experienced a 0.49 °C (95%CI (0.09, 0.89)) and a 1.74 °C (95%CI (1.09, 2.38)) lower daily mean HI[individual] during intervention days, respectively (Table 3). Urban OutWor experienced a 6.60 °C (95%CI (4.11, 9.09)) lower daily max HI[individual] during the intervention (Table 4).

Overall, participants had lower daily mean or max HI[individual] on intervention days during weekends compared to intervention days on weekdays (Supplemental File 8). ITT results and PP results were similar, with slightly smaller estimated effect sizes in PP (β estimate of intervention −0.59 (95%CI (−0.88, −0.30)) in ITT vs. −0.49 (95%CI (−0.79, −0.20)) in PP on daily mean of individually experienced HI, and −1.40 (95%CI (−2.27, −0.53)) in ITT vs. −0.99 (95%CI (−1.90, −0.08)) in PP on daily max of individually experienced HI) (Supplemental File 8). Outlier removal minimally affected the intervention effect on daily mean HI difference (β estimate of intervention −0.59 (95%CI (−0.88, −0.30)) in ITT vs. −0.51 (95%CI (−0.83, −0.19)) in ITT with no outlier removal). Outlier removal affected the intervention effect on daily max HI difference more (β estimate of intervention −1.40 (95%CI (−2.27, −0.53)) in ITT vs. −0.58 (95%CI (−1.93, 0.76)) in ITT with no outlier removal) (Supplemental File 9).

Body measurement change ratios (%) are shown in Table 5. Overall, participants had a small decrease in weight, body fat and muscle mass and a small increase in body water. These change ratios were only statistically significant in participants who were obese. A −0.29% (95%CI (−0.45, −0.13)) weight change ratio is equivalent to a 0.52 lb. (95%CI (0.23, 0.81)) weight loss for a participant weighing 180 lbs. at baseline. There was no significant difference in body measurement change ratios in sensitivity analysis including participants with extreme measurement change ratios (Supplemental File 10).

## 4. Discussion

This study investigates whether spending an additional 30 min outdoors daily in summer is feasible in an urban versus rural environment, and if it changes daily steps and individually experienced HI of participants. Rain, heat, and time conflicts were leading factors hindering participants from spending an additional 30 min outdoors in both environments. This result is consistent with findings in previous studies [1,2], suggesting heat is a barrier for physical activity in summer. Since it is hot and humid with frequent storms in the summer in the southeastern states of the U.S., heat and rain may be barriers to outdoor time and associated physical activity benefits. We found participants who self-identified as physically inactive had a 15.70% (95%CI (8.94%, 22.46%)) lower probability of intervention compliance. These results indicate participants starting with less physical exercise might perceive higher barriers to spending time outdoors, suggesting efforts to improve time spent outdoors among participants with less physical exercise may require initially reducing the amount of time (e.g., start with 15 min) or other methods of encouraging behavior change.

Participants increased daily steps by 637 (95%CI (83, 1192)) on intervention days. This relation was driven by increased daily steps in Rural residents, who walked a mean of 1063 (95%CI (273, 1851)) more steps (baseline daily 4346 steps, 24% increase) on intervention days. In contrast, Urban OutWor participants, with much higher baseline steps, only had a small increase in daily steps on intervention days. The results suggested that the benefits of the increased time spent outdoors may be more significant in physically less-active participants. The built environment (e.g., sidewalks, trails, recreational facilities) impacts physical activity [45,46,47,48]. Birmingham is the second most walkable city in AL while Wilcox County is considered a car-dependent, less walkable location, based on the walk score metric [49]. Among participants, Birmingham was rated as a more activity-friendly, walkable location with more access to recreational facilities compared to Wilcox County in the PANES results, although some neighborhood environment variables in the PANES may not be relevant for rural neighborhoods [30,50]. These differences in the built environment could at least partially explain differences in neighborhood-level microclimates and might impact the intervention effects on promoting physical activity among participants. The generalizability of the results presented to other populations with similar or different demographics should be evaluated in future studies. Spending an additional 30 min outdoors daily is minimally limited by socioeconomic status (SES), although we acknowledged that conflicts of time/limited free time associated with lower SES from participants were reported (Figure 2). We believe our results may be useful to provide an additional intervention method to promote physical activity among populations with similar SES in both urban and rural settings, especially in subtropical/tropical states in the U.S. Small but significant changes in body measurement change ratios were detected among participants who were obese after participation, suggesting the intervention benefits may be more significant among people with higher BMI.

While previous studies use weather station data to estimate the effect of ambient conditions on physical activity [3,51,52,53], in the current study we have additionally measured microclimates experienced by participants within urban and rural neighborhoods and individual HI experienced by participants as they move through outdoor and indoor environments. This is important as previous studies have shown a wide variation in temperature and humidity experienced within cities, suburban, and rural environments [54,55,56,57,58,59]. Overall, participants experienced lower daily mean or max HI on intervention days after accounting for ambient conditions, suggesting the additional 30 min outdoors did not result in increased heat exposure. Urban and Rural participants experienced a similar small change in daily mean or max HIs on intervention and baseline days, while outdoor workers had significantly reduced HI exposure during intervention days. Outdoor workers may have carried out the intervention in the cool hours of the day, thereby reducing their overall daily heat index exposure. Since participants were free to choose the time of day to spend the additional 30 min outdoors, we think most of the participants carried out the intervention either on early mornings or after sunset to avoid the hottest hours. Additionally, because the estimated prevalence of home central air-conditioning was not high for participants, outdoor environment may be cooler than homes when participants carried out the intervention, leading to reduced individually experienced HI. However, there was high missingness for the central air-conditioning response, so it is difficult to draw conclusions.

Two baseline days were weekdays while two out of five intervention days were weekends. To remove the weekend effect, we compared the daily mean or max individually experienced HI on baseline days vs. intervention on weekdays. However, this step considerably reduced the observation sample size. We observed that weekends augmented the negative association between the intervention and daily mean or maximum individually experienced HI in participants. Our results show that non-outdoor worker participants increased daily steps during the weekend but did not increase individually experienced heat indexes.

To address the concern that the thermometer on the shoe might pick up high temperatures due to artifacts (e.g., close to warm surfaces) when the actual environment was not hot, we removed upper outliers. The removal had minimal impact on the intervention effect on daily mean HI[individual]. Pedometer data imputation changed the intervention effect substantially.

In future studies, researchers may use pedometers with built-in daily reading features, or accelerometers to monitor physical activity more accurately. The benefits of additional time spent outdoors would likely include increased physical activity and may be more pronounced after longer term compliance, although this requires further study. Using advanced wearable technologies (e.g., FitBit, Apple Watch), albeit more expensive, to incorporate heart rate, time spent in different intensity activity, energy expenditure and total distance to measure physical activity more accurately would be an important next step to quantify the physical activity benefits. Participants could be further encouraged to engage in physical activity from these additional real-time feedback measures. Benefits beyond improved physical activity, such as improved mental health, an improved sense of well-being and blood pressure etc. suggested by previous studies could also be included [60,61,62].

## 5. Conclusions

In conclusion, providing a nudge to spend a small amount of additional time outdoors daily with pedometer visual feedbacks may be a feasible intervention to promote physical activity. The current study additionally suggests that outdoor ambient conditions at neighborhood level, in addition to regional weather station measurements, are an important factor in determining physical activity in both urban and rural environments in summer months. Finally, our study results indicating a stronger intervention effect in the rural environment suggest further study of differences in built environment characteristics across urban and rural landscapes is warranted.

## Figures and Tables

**Table 1:** Participant demographics and characteristics.

Parameters	Urban OutWor	*p*-Value(1) ^a^	Urban Residents	*p*-Value(2) ^a^	Rural Residents
Participant number	32	NA	57	NA	88
Median age (range), years	39.5 (21–60)	0.03 *	45 (20–69)	0.17	54 (19–67)
Gender: Female	32 (100%)	NA	57 (100%)	NA	88 (100%)
% Black or African American	30 (94%)	NA	55 (96%)	NA	88 (100%)
Employed	32 (100%)	NA	34 (60%)	0.04 *	37 (42%)
Central air conditioning at home		0.42 ^b^		0.04 ^b,^*	
Yes	12 (38%)		36 (63%)		21 (24%)
No	6 (19%)		11 (19%)		17 (19%)
Missing data	14 (44%)		10 (18%)		50 (57%)
Education		0.52 ^b^		0.61 ^b^	
≤High School Diploma (or Equivalence)	14 (44%)		29 (51%)		40 (45%)
>High School Diploma (or Equivalence)	18 (56%)		28 (49%)		46 (52%)
Missing data	0 (0%)		0 (0%)		2 (3%)
Annual household income		0.80 ^b^		0.90 ^b^	
<USD 20,000	22 (69%)		37 (65%)		57 (65%)
≥USD 20,000	10 (31%)		19 (33%)		28 (32%)
Missing data	0 (0%)		1 (2%)		3 (3%)
Body mass index (BMI) (median, range) from check-in session	34.3(19.3–52.3)	0.19	35.8 (24.7–60.3)	0.57	36.6 (19.5–64.8)
Obesity prevalence		0.85^c^		0.52 ^c^	
Overweight (BMI ≥25 and <30) from check-in session	6 (19%)		11 (19%)		13 (15%)
Obese (BMI ≥30.0) from check-in session	22 (69%)		45 (79%)		71 (81%)
Body fat % (median, range) from check-in session	42.4 (22.9–52.8)	0.04 *	45.2 (25.7–54.7)	0.04 *	47.3 (25.3–70.6)
Body water % (median, range) from check-in session	41.4 (35.0–52.5)	0.02 *	39.0 (33.3–52.8)	0.02 *	38.0 (19.4–51.8)
Godin activity level		0.20		0.29	
Active	27 (38%)		53 (93%)		77(88%)
Insufficient active	5 (16%)		4 (7%)		11 (13%)
Diabetic		0.98		0.0004 *	
Yes	4 (12%)		7 (12%)		35 (40%)
No	28 (88%)		50 (88%)		53 (60%)
Self-reported health condition		0.62 ^d^		0.29 ^d^	
Good	25 (78%)		47 (82%)		63 (72%)
Fair	6 (19%)		9 (16%)		19 (22%)
Poor	1 (3%)		1 (2%)		2 (2%)
Missing	0 (0%)		0 (0%)		4 (5%)

Note: *p*-values were obtained from Welch two sample t-test for continuous variables or Pearson’s Chi-square test for categorical variables. “*” denotes a statistically significant difference with *p*-values < 0.05. ^a^ *p*-value(1) was obtained from comparison between Urban residents and Urban Outdoor Workers (Urban OutWor), *p*-value(2) was obtained from comparison between Urban residents and Rural residents in the same category. ^b^ Chi-squared test for available data only. ^c^ Chi-squared test for obese vs. non-obese. ^d^ Chi-square test for good vs. less than good.

**Table 2:** Daily pedometer steps on baselines days and intervention days.

Group	All	Rural Residents	Urban Residents	Urban OutWor
Fixed effect	β 95%CI	β 95%CI	β 95%CI	β 95%CI
Intercept	12188 (2304, 22069)	8001 (−6564, 22566)	11120 (−9665, 31906)	23100 (−1962, 48133)
Intervention	637 (83, 1192) *	1063 (273, 1851) *	167 (−828, 1161)	222 (−1163, 1601)
Weather Station (WS) Heat Index (HI) Max (°C)	−244 (−566, 78)	−94 (−701, 513)	−313 (−900, 276)	−377 (−1283, 527)
Neighborhood HI Max (°C)	58 (−115, 231)	193 (−52, 442)	16 (−298, 326)	−71 (−550, 407)
WS HI Mean (°C)	44 (−368, 456)	101 (−481, 684)	118 (−669, 908)	−494 (−1882, 894)
Neighborhood HI Mean (°C)	34 (−314, 381)	−260 (−790, 270)	47 (−642, 732)	618 (−431, 1667)
WS rain	−479 (−1084, 125)	−686 (−1458, 87)	367 (−921, 1654)	92 (−2070, 2244)

Note: “*” denotes a 95% confidence interval (CI) does not contain 0. Results were Intent-to-Treat. The model for all participants did not include a group factor. Models were adjusted for participant age, education level, annual household income level, employment, measured body fat (%), being diabetic, activity level in Godin questionnaire, and self-reported health condition.

**Table 3:** Intervention effects on daily mean heat index (°C) experienced by individuals in different population groups.

Group	All	Rural Residents	Urban Residents	Urban OutWor
Fixed effect	β 95%CI	β 95%CI	β 95%CI	β 95%CI
Intercept	24.45 (19.23, 29.67)	24.47 (15.99, 32.96)	31.58 (22.02, 41.12)	5.43 (−7.78, 18.63)
Intervention	−0.59 (−0.88, −0.30) *	−0.49 (−0.89, −0.09) *	−0.28 (−0.80, 0.24)	−1.74 (−2.38, −1.09) *
WS HI Mean (°C)	NA	NA	NA	0.95 (0.59, 1.31) *
Neighborhood HI Mean (°C)	0.21 (0.09, 0.34) *	0.01 (−0.19, 0.22)	0.04 (−0.17, 0.25)	NA
Wind speed Mean (m/s)	0.69 (0.23, 1.15) *	0.93 (0.22, 1.64) *	0.56 (−0.16, 1.29)	1.51 (0.39, 2.63) *
WS Rain	0.24 (−0.08, 0.55)	0.32 (−0.06, 0.69)	0.03 (−0.62, 0.67)	0.06 (−0.80, 0.92)

Note: “*” indicates a 95% confidence interval does not contain 0. NA in neighborhood HI or WS HI daily mean indicates that neighborhood HI or WS HI daily mean was not included in the model selection based on Akaike Information Criterion (AIC). Models were adjusted for participant age, annual household income level, education level, measured body fat (%), log(mean daily steps), employment, being diabetic, self-reported health condition, and Godin activity level. Models for all participants did not include a group factor.

**Table 4:** Intervention effects on daily maximum heat index (°C) experienced by individuals in different population groups.

Group	All	Rural Residents	Urban Residents	Urban OutWor
Fixed effect	β 95%CI	β 95%CI	β 95%CI	β 95%CI
Intercept	45.13 (33.44, 56.73)	34.91 (8.80, 51.01)	55.64 (34.37, 76.79)	−10.00 (−59.85, 39.87)
Intervention	−1.40 (−2.27, −0.53) *	−0.24 (−1.36, 0.88)	−0.73 (−2.32, 0.86)	−6.60 (−9.09, −4.11) *
WS HI Max (°C)	NA	NA	NA	2.03 (0.71, 3.35) *
Neighborhood HI Max(°C)	0.15 (−0.07, 0.37)	0.07 (−0.21, 0.36)	−0.01(−0.43, 0.41)	NA
Wind speed Max (m/s)	0.49 (0.21, 0.76) *	0.23 (−0.13, 0.59)	1.01 (0.53, 1.49) *	0.30 (−0.60, 1.19)
WS Rain	−0.05 (−0.98, 0.88)	1.01 (0.02, 2.00) *	−2.30 (−4.22, −0.39) *	−3.52 (−6.74, −0.30) *

Note: “*” indicates a 95% confidence interval does not contain 0. NA in neighborhood HI or WS HI daily mean indicates that neighborhood HI or WS HI daily mean was not included in the model selection based on AIC. Models were adjusted for participant age, annual household income level, education level, measured body fat (%), log(mean daily steps), employment, being diabetic, self-reported health condition, and Godin activity level. Models for all participants did not include a group factor.

**Table 5:** Body measurement change ratios (%) of participants after the intervention.

Body Measurement Change Ratio (%) ^a^	Mean (95%CI)	Participant N	Obese Level ^b^	Mean (95%CI)	Participant N
Weight	−0.29 (−0.45, −0.13) *	176	Normal	−0.33 (−1.26, 0.6)	9
			Overweight	−0.3 (−0.75, 0.15)	30
			Obese	−0.28 (−0.46, −0.11) *	137
Body fat	−1.11 (−1.73, −0.49) *	167	Normal	−0.92 (−3.56, 1.71)	9
			Overweight	−1.15 (−2.31, 0.004)	28
			Obese	−1.12 (−1.85, −0.38) *	130
Body water	0.86 (0.34, 1.38) *	167	Normal	0.47 (−1.2, 2.15)	9
			Overweight	0.65 (−0.14, 1.44)	28
			Obese	0.93 (0.3, 1.57) *	130
Muscle mass	−0.87 (−1.42, −0.32) *	167	Normal	−0.42 (−2.39, 1.55)	9
			Overweight	−0.78 (−1.78, 0.22)	28
			Obese	−0.93 (−1.59, −0.27) *	130

Note: “*” denotes a 95% confidence interval does not contain 0. ^a^ Body measurement change ratio = (body measurement after intervention—body measurement before intervention)/body measurement before intervention × 100%. ^b^ Obese level: Normal = (BMI < 25), Overweight = (BMI ≥ 25 and < 30), Obese = (BMI ≥ 30) [44].

## Supplementary Materials

The following are available online at https://www.mdpi.com/1660-4601/17/20/7558/s1, Supplemental File 1. Data collection and processing flowcharts. Supplemental File 2. Results of risk difference regression describing the relation between the probability of intervention compliance and ambient conditions, individual-level factors. Supplemental File 3. Full results of the linear mixed models describing the relation of the intervention and daily pedometer steps with an interaction term between intervention and groups in Intent-to-Treat (ITT). Supplemental File 4. Results of linear mixed effects models describing the relation between the intervention (weekdays vs. weekend) and the daily pedometer steps in Intent-to-Treat (ITT). Supplemental File 5. Results of linear mixed models describing the relation between the intervention and the daily pedometer steps in Per-Protocol (PP). Supplemental File 6. Effect of data processing methods on the pedometer step results. Supplemental File 7. Results of linear mixed models describing the relation between the intervention and the daily mean or max heat index experienced by individuals with an interaction term between intervention and groups in ITT. Supplemental File 8. Sensitivity analysis of intervention (or intervention and weekdays vs. intervention and weekend) effect on HI[individual] in ITT and PP. Supplemental File 9. Effect of ITT outlier removal on daily mean and max HI difference (°C) between HI[individual] and HI[WS]. Supplemental File 10. Body measurement change ratios of supplemental datasets including extreme body measurement change ratios.
