# Supplementary material for: Effect of an Additional 30 Minutes Spent Outdoors during Summer on Daily Steps and Individually Experienced Heat Index
Source: Int J Environ Res Public Health. 2020 Oct 17;17(20):7558. doi: 10.3390/ijerph17207558 (PMC7589302; doi:10.3390/ijerph17207558)
Supplement: Supplementary file 1 [file ijerph-17-07558-s001.zip › Supplementary files IJERPH.pdf]

Supplemental Files

of

Effect of time spent outdoors during summer on daily steps and individually experienced heat index

List of Supplemental Files

Supplemental File 1. Data collection and processing flowcharts. pdf

Supplemental File 2. Results of risk difference regression describing the relation between the probability of intervention compliance and ambient conditions, individual-level factors. pdf

Supplemental File 3. Full results of the linear mixed models describing the relation of the intervention and daily pedometer steps with an interaction term between intervention and groups in Intent-to-Treat (ITT). pdf

Supplemental File 4. Results of linear mixed effects models describing the relation between the intervention (weekdays vs. weekend) and the daily pedometer steps in Intent-to-Treat (ITT). pdf

Supplemental File 5. Results of linear mixed models describing the relation between the intervention and the daily pedometer steps in Per-Protocol (PP). pdf

Supplemental File 6. Effect of data processing methods on the pedometer step results. pdf

Supplemental File 7. Results of linear mixed models describing the relation between the intervention and the daily ***mean or max*** heat index experienced by individuals with an interaction term between intervention and groups in ITT. pdf

Supplemental File 8. Sensitivity analysis of intervention (or intervention &weekdays vs. intervention &weekend) effect on HI[individual] in ITT and PP. pdf

Supplemental File 9. Effect of ITT outlier removal on daily mean and max HI difference(°C) between HI[individual] and HI[WS]. Pdf

Supplemental File 10. Body measurement change ratios of supplemental datasets including extreme body measurement change ratios. pdf

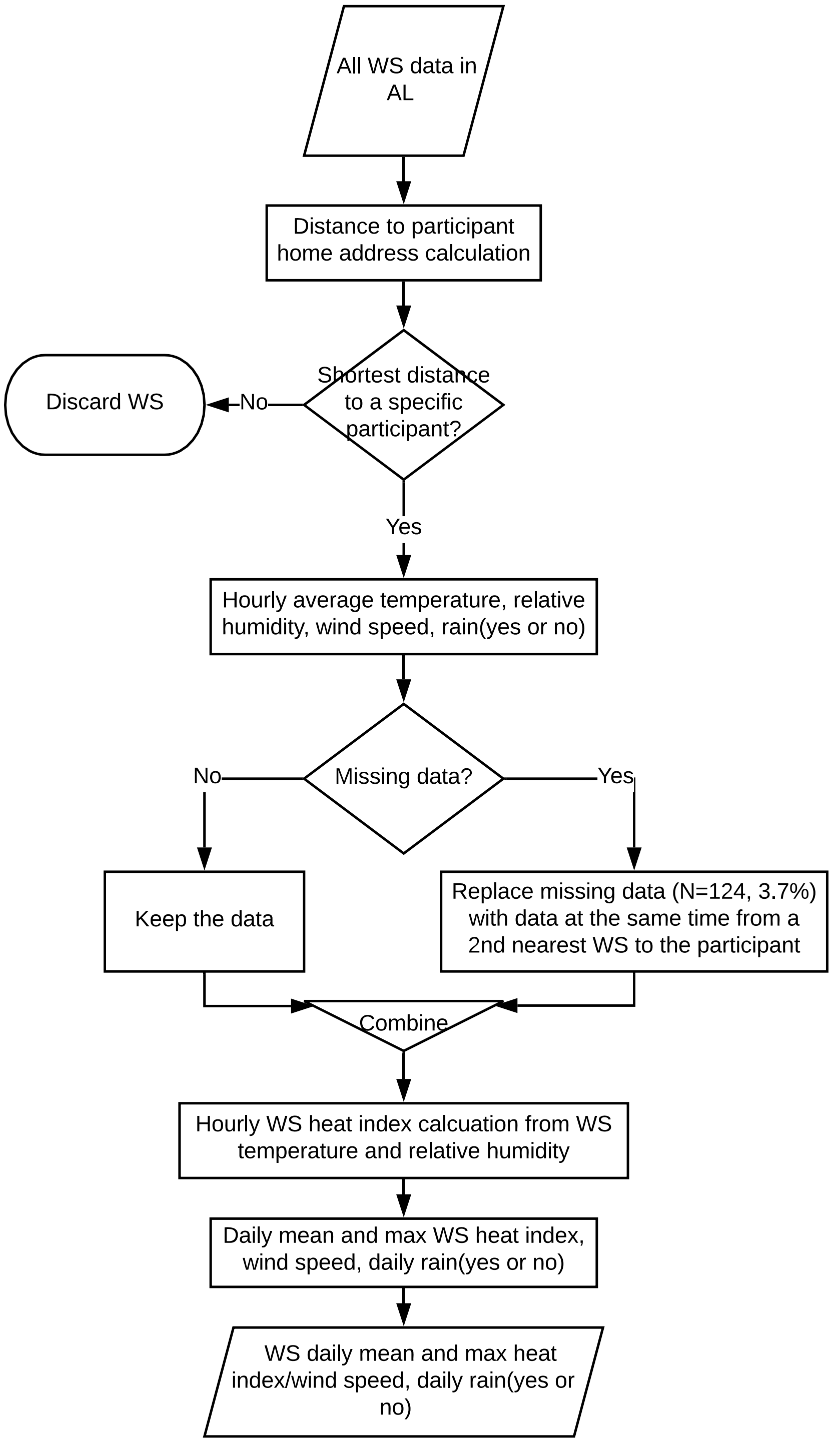

Supplemental File 1. Data collection and processing flowchart. Bessemer Airport WS and Birmingham International Airport WS were closest to participants' residences in Birmingham while Craig Field WS, Demopolis Municipal Airport WS, Mac Crenshaw Memorial Airport WS, and Middleton Field Airport WS were closest to participants' residences in Wilcox County.

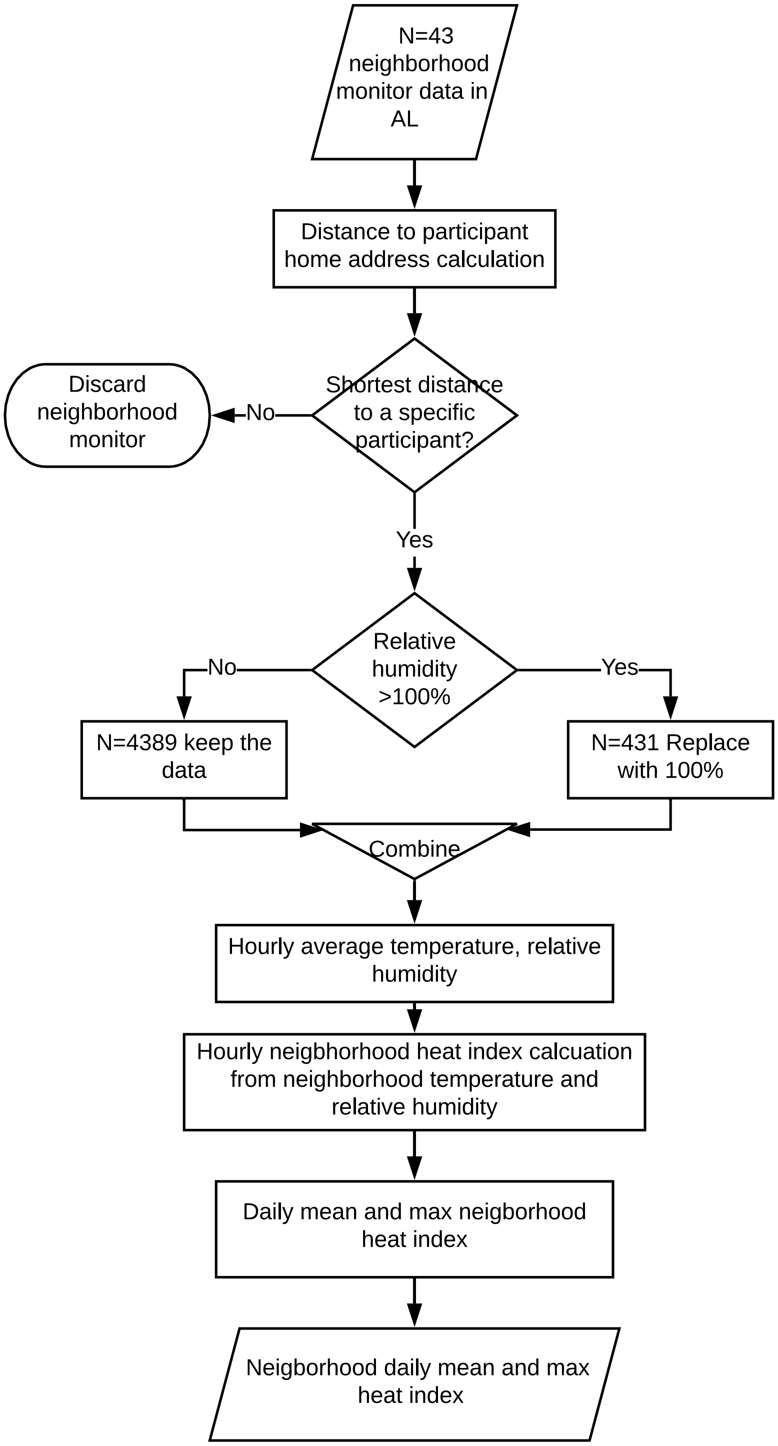

Supplemental File 1 (continued). Data collection and processing flowchart.

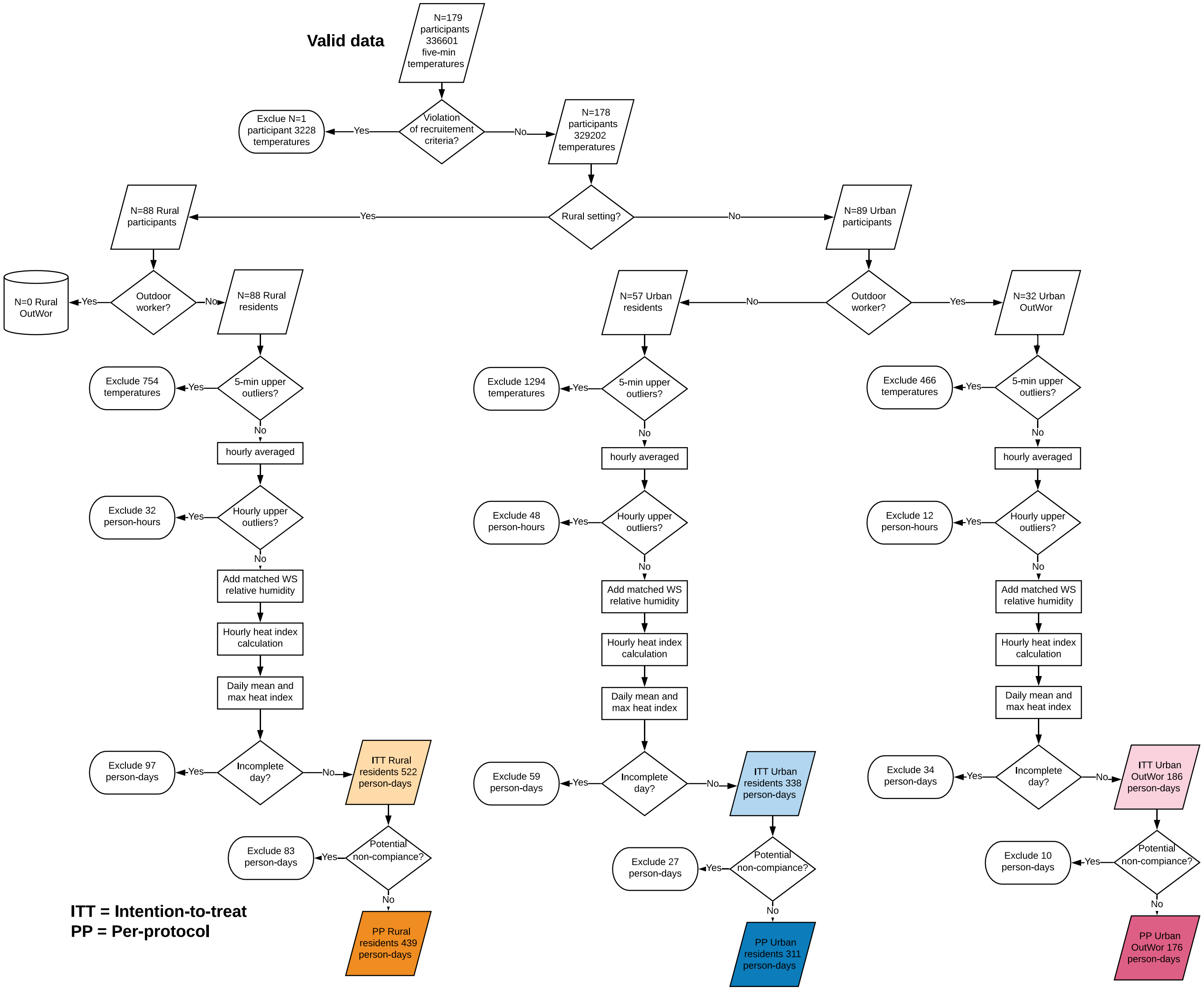

Supplemental File 1 (continued). Data collection and processing flowchart.

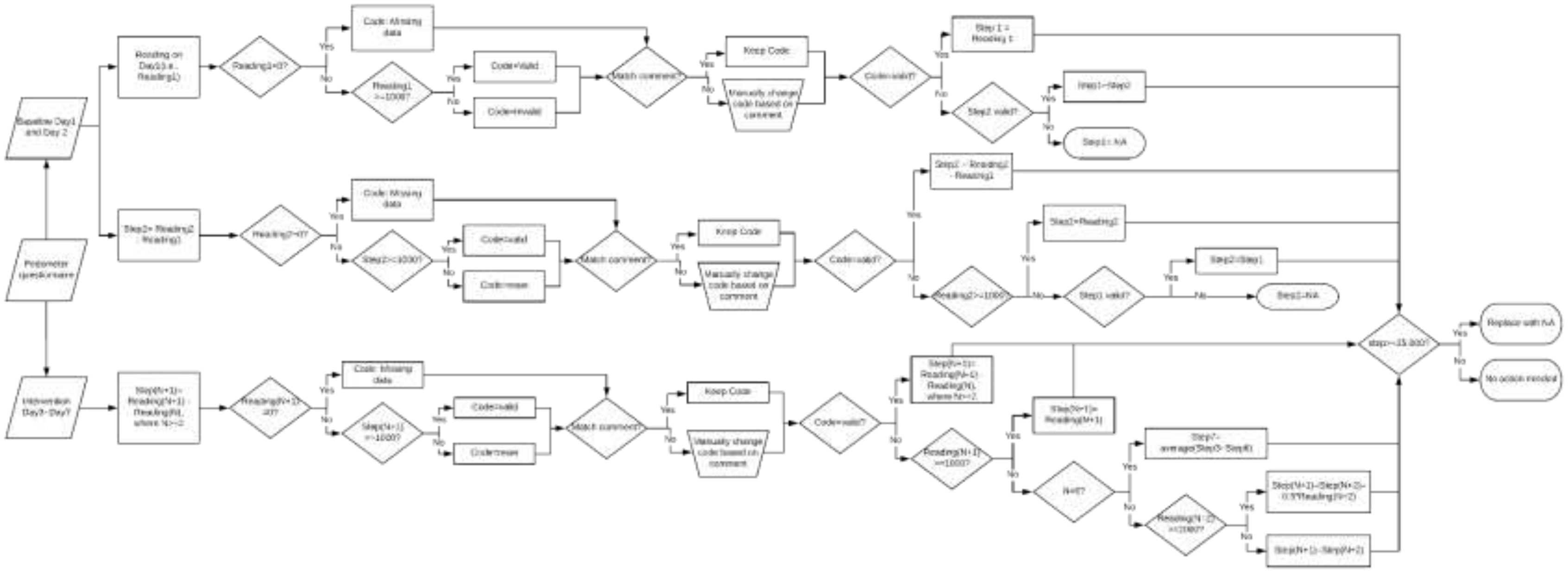

Supplemental File 1 (continued). Data collection and processing flowchart.

Body measurement category	Participant N with available data	Participant N removed due to unrealistic or extreme body measurement change	Participant N remained in the main results
Weight (lbs.)	177	0	177
Body fat (%)	172	5	167
Body water (%)	172	5	167
Muscle mass (lbs.)	172	5	167

Supplemental File 1 (continued). Body measurement processing record. The body fat, body water, muscle mass changes of five participants considered unrealistic or extreme were excluded (e.g., participants had body fat change ratio of 17-44%). The exclusion effect was examined in sensitivity analysis.

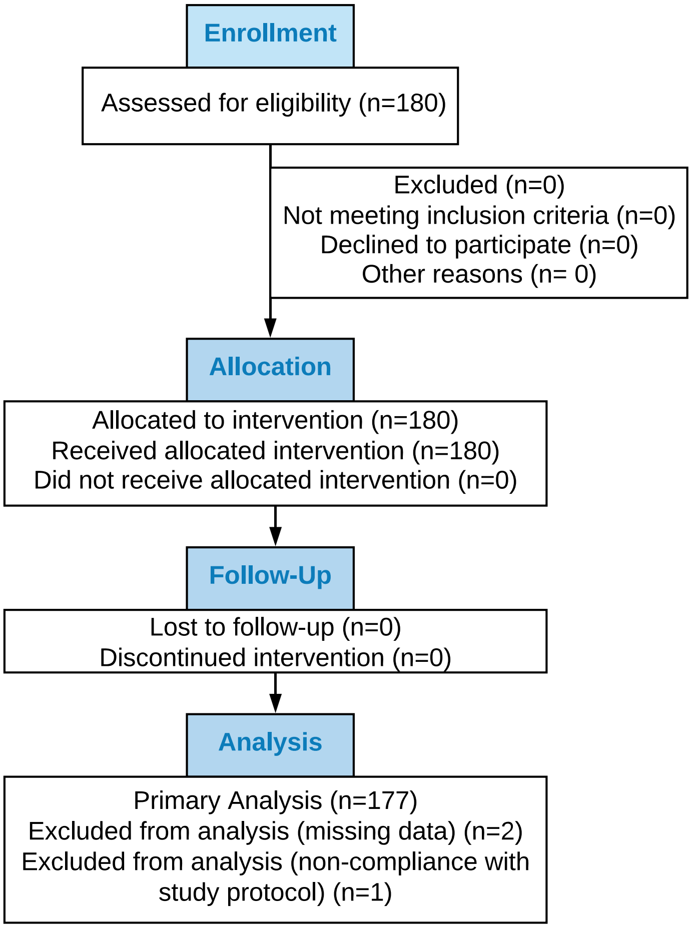

Supplemental File 1 (continued). CONSORT flowchart.

Supplemental File 2. Results of risk difference regression describing the relation between the probability of intervention compliance and ambient conditions, individual-level factors.

Fixed effect	β 95% CI in percent (%)
Intercept	-99.73 (-266.22, 66.77)
$HI[individual]_{daily\ mean}$ (°C)	0.95 (-0.11, 2.02)
$HI[WS]_{daily\ mean}$ (°C)	3.28 (-2.19, 8.75)
$HI[neighborhood]_{daily\ mean}$ (°C)	0.3 (-3.84, 4.45)
$HI[individual]_{daily\ max}$ (°C)	0.47 (0.01, 0.92)*
$HI[WS]_{daily\ max}$ (°C)	0.34 (-3.52, 4.19)
$HI[neighborhood]_{daily\ max}$ (°C)	0.07 (-1.90, 2.03)
Wind Spd daily mean (m/s)	4.51 (-6.47, 15.49)
Weekend	2.41 (-4.64, 9.46)
Age	0.17 (-0.09, 0.43)
Education > high school	5.02 (-2.3, 12.35)
Annual household income >20k	2.36 (-5.52, 10.24)
Rural residents[a]	6.4 (-3.92, 16.71)
Urban OutWor[a]	8.75 (-1.7, 19.2)
Body fat (%)	-0.53 (-1.13, 0.08)
Diabetic	-6.9 (-16.2, 2.39)
Health condition in Fair[b]	-6.37 (-17.2, 4.46)
Health condition in Poor[b]	-0.18 (-15.23, 14.87)
Godin Inactivity	-15.70 (-22.46, -8.94)*
Log(daily steps)	3.43 (-0.38, 7.23)
Rain	9.1 (1.5, 16.71)*

Note: ‘*’ indicates a 95% confidence interval does not contain 0.

[a]Compared to Urban participants

[b]Compared to health condition in Good

Supplemental File 3. Results of the linear mixed models describing the relation of the intervention and daily pedometer steps with an interaction term between intervention and groups in Intent-to-Treat (ITT).

Population	All
Fixed effects	β 95%CI
Intercept	11415 (1164, 21667)
WS HI Max(°C)	-164 (-494, 167)
Neighborhood HI Max(°C)	57 (-117, 231)
WS HI Mean(°C)	-1 (-415, 413)
Neighborhood HI Mean(°C)	-2 (-353, 349)
WS wind speed mean (m/s)	411 (-545, 1367)
WS rain	-493 (-1098, 111)
Age	16 (-13, 44)
Annual household income >20k	-74 (-865, 719)
Education > high school	-130 (-864, 601)
Employed	414 (-406, 1233)
Body fat (%)	-86 (-143, -29)*
Diabetic	329 (-554, 1210)
Godin Inactivity	-199 (-909, 508)
Health condition in Fair[a]	499 (-416, 1408)
Health condition in Poor[a]	316 (-1954, 2586)
Intervention	250 (-697, 1196)
Rural residents	-417 (-1639, 802)
Urban OutWor	1067 (-362, 2498)
Intervention*Rural residents	912 (-321, 2145)
Intervention*Urban OutWor	-325 (-1779, 1128)

Note: ‘*’ denotes a 95% confidence interval does not contain 0. The model for all participants did not include a group factor. Urban resident was the reference group.

[a]Compared to health condition in Good

Supplemental File 4. Intervention &weekdays vs. intervention &weekend effect on daily pedometer steps in ITT.

Model	Group	All	Rural residents	Urban residents	Urban OutWor
1	Fixed effect	β 95%CI	β 95%CI	β 95%CI	β 95%CI
	Intercept	8968 (-2325, 20246)	1823 (-15505, 19120)	10282 (-15117, 35711)	22308 (-7548, 52197)
	Intervention &weekdays	484 (-128, 1095)	773 (-129, 1675)	152 (-881, 1184)	189 (-1351, 1727)
	Intervention &weekends	886 (190, 1580)*	1428 (464, 2389)*	232 (-1276, 1737)	290 (-1693, 2262)
2	Fixed effect	β 95%CI	β 95%CI	β 95%CI	β 95%CI
	Intercept	5421 (-10731, 21572)	9596 (-11764, 30964)	2706 (8985, 64397)	5526 (-108772, 120406)
	Weekend	453 (-344, 1250)	318 (-837, 1475)	593 (-1887, 3073)	1423 (-3495, 6292)

Note: ‘*’ denotes a 95% confidence interval does not contain 0. Models for all participants did not include a group factor. Model 2 includes data on intervention days only.

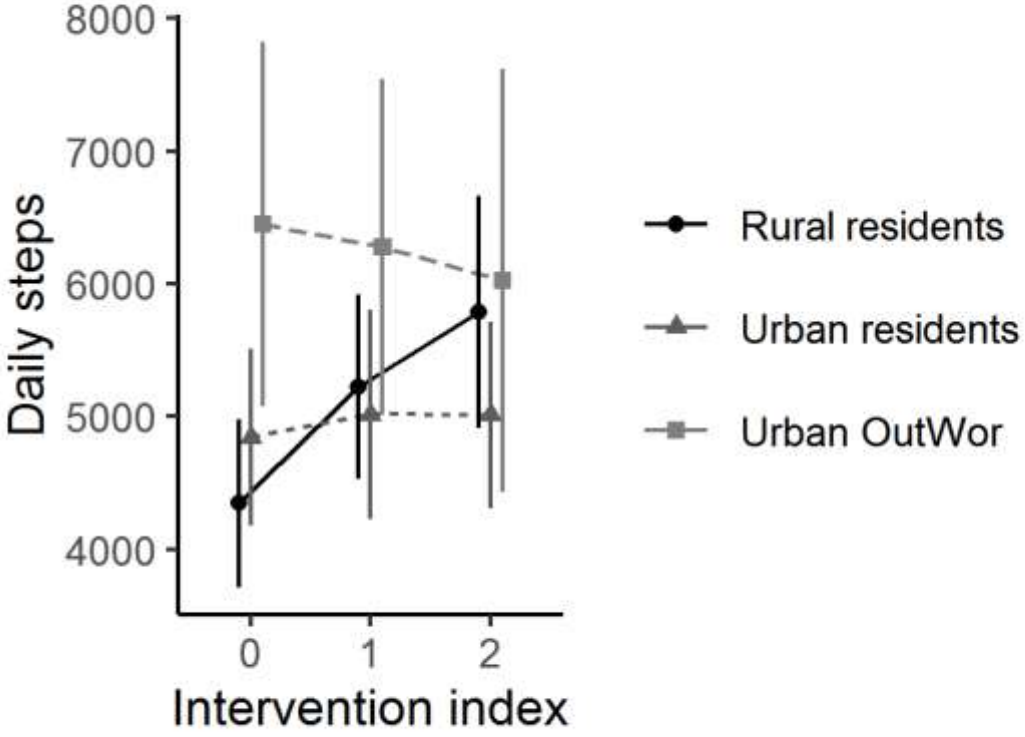

Supplemental File 4 (continued). Intervention &weekdays vs. intervention &weekend effect on daily pedometer steps. The population mean of the individual mean daily steps on baseline days (intervention index = 0), intervention &weekdays (intervention index =1) and intervention &weekend (intervention index = 2) in different population groups. The 95% confidence intervals are shown.

Supplemental File 5. Results of linear mixed models describing the relation between the intervention and the daily pedometer steps in Per-Protocol (PP).

Population	All	Rural residents	Urban residents	Urban OutWor
Fixed effects	β 95%CI	β 95%CI	β 95%CI	β 95%CI
Intercept	13143 (2754, 23529)	9727 (-5926, 25378)	5624 (-15840, 27089)	25303 (-146, 50729)
WS HI Max(°C)	-279 (-615, 56)	-76 (-725, 573)	-375 (-977, 227)	-235 (-1164, 694)
Neighborhood HI Max(°C)	31 (-149, 211)	125 (-121, 375)	112 (-210, 433)	-94 (-591, 398)
WS HI Mean(°C)	43 (-392, 479)	100 (-521, 721)	284 (-539, 1107)	-572 (-1986, 845)
Neighborhood HI Mean(°C)	83 (-287, 454)	-237 (-790, 314)	-25 (-736, 685)	523 (-558, 1603)
WS wind speed mean (m/s)	648 (-360, 1656)	100 (-1367, 1555)	1332 (-405, 3068)	599 (-2501, 3701)
WS rain	-185 (-834, 465)	-279 (-1121, 565)	577 (-748, 1902)	-247 (-2494, 1990)
Age	13 (-16, 42)	16 (-21, 54)	13 (-22, 49)	-31 (-172, 111)
Annual household income >20k	-178 (-962, 610)	120 (-963, 1207)	-778 (-2003, 450)	-1133 (-3484, 1227)
Education > high school	-89 (-831, 653)	951 (-63, 1961)	-353 (-1491, 789)	-2486 (-4559, -389)*
Employed	602 (-189, 1392)	201 (-844, 1241)	655 (-379, 1689)	NA
Body fat (%)	-105 (-161, -50)*	-88 (-168, -7)*	-40 (-126, 44)	-116 (-254, 21)
Diabetic	540 (-352, 1426)	60 (-1027, 1135)	337 (-956, 1631)	2977 (-1112, 7026)
Godin Inactivity	-179 (-898, 533)	-1452 (-2466, -442)*	375 (-631, 1375)	2103 (103, 4114)*
Health condition in Fair[a]	468 (-481, 1412)	912 (-348, 2155)	634 (-544, 1814)	-1348 (-4203, 1455)
Health condition in Poor[a]	557 (-1768, 2887)	4116 (703, 7472)*	-2983 (-6177, 223)	-313 (-5544, 4911)
Intervention	579 (5, 1154)*	958 (130, 1786)*	229 (-796, 1255)	57 (-1358, 1471)

Note: ‘*’ denotes a 95% confidence interval does not contain 0. The model for all participants did not include a group factor.

[a]Compared to health condition in Good

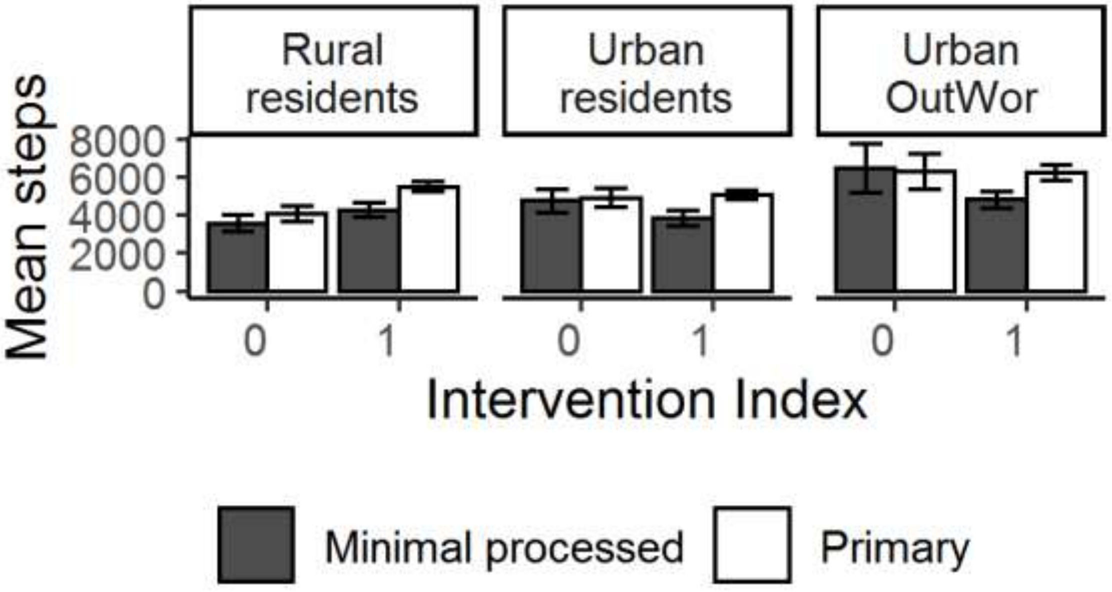

Supplemental File 6. Effect of data processing methods on the pedometer step results. Primary dataset was obtained by following the decision tree in Supplemental File 1. Minimal processed dataset was obtained by replacing the negative steps with NA. The 95% confidence intervals are shown.

Supplemental File 6 (continued). Effect of data processing methods on the pedometer step results. Results of linear mixed models describing the relation between the intervention and the daily pedometer steps processed in ***minimal processed*** dataset.

Population	All	Rural residents	Urban residents	Urban OutWor
Fixed effects	β 95%CI	β 95%CI	β 95%CI	β 95%CI
Intercept	6540 (-5762, 18838)	-1189 (-19451, 17073)	9597 (-19878,39091)	4085 (-19227,27342)
WS HI Max(°C)	-187 (-587, 213)	594 (-166, 1352)	-146 (-1013,725)	-732 (-1591,125)
Neighborhood HI Max(°C)	28 (-190, 245)	7 (-311, 326)	325 (-132,776)	-52 (-519,416)
WS HI Mean(°C)	607 (91, 1123)*	129 (-602, 861)	1001 (-162,2168)	957 (-346,2257)
Neighborhood HI Mean(°C)	-352 (-783, 78)	-540 (-1206, 121)	-1309 (-2306,-316)*	96 (-863,1055)
WS wind speed mean (m/s)	286 (-886, 1458)	-313 (-1999, 1374)	302 (-2163,2767)	1796 (-992,4572)
WS rain	-122 (-872, 628)	271 (-686, 1227)	-865 (-2710,982)	669 (-1301,2623)
Age	27 (-9, 64)	22 (-29, 73)	49 (-3,101)	-1 (-150,146)
Annual household income >20k	-477 (-1459, 506)	-139 (-1617, 1336)	-1761 (-3562,31)	-1194 (-3654,1303)
Education > high school	-65 (-1000, 867)	-216 (-1595, 1166)	1430 (-283,3166)	-1707 (-3887,472)
Employed	578 (-410, 1569)	163 (-1262, 1595)	1096 (-375,2575)	NA
Body fat (%)	-110 (-180, -40)*	-95 (-207, 17)	-119 (-241,4)	-49 (-190,93)
Diabetic	579 (-527, 1684)	-36 (-1496, 1424)	2550 (574,4495)*	1505 (-2841,5798)
Godin Inactivity	-74 (-976, 827)	-991 (-2383, 392)	254 (-1225,1757)	2121 (25,4219)
Health condition in Fair[a]	-353 (-1516, 809)	-530 (-2210, 1149)	1097 (-640,2854)	-2213 (-5013,584)
Health condition in Poor[a]	1195 (-1833, 4209)	4586 (-291, 9364)	-1973 (-6711,2789)	-103 (-5677,5433)
Intervention	-271 (-960, 418)	427 (-544, 1397)	-972 (-2392,447)	-976 (-2233,276)

Note: '*' denotes a 95% confidence interval does not contain 0. The model for all participants did not include a group factor.

[a]Compared to health condition in Good

Supplemental File 7. Results of linear mixed models describing the relation between the intervention and the daily *mean or max* heat index experienced by individuals with an interaction term between intervention and groups in ITT.

	Model 1		Model2
Dependent variable	HI[individual] ***Mean***	Dependent variable	HI[individual] ***Max***
Fixed effects	β 95%CI	Fixed effects	β 95%CI
(Intercept)	23.39 (18.03, 28.75)*	Intercept	40.4 (28.47, 52.26)*
Neighborhood Mean HI(°C)	0.22 (0.09, 0.34)*	Neighborhood Max HI(°C)	0.21 (-0.02, 0.43)
WS Mean HI(°C)	NA	WS Max HI(°C)	NA
WS wind speed mean (m/s)	0.68 (0.23, 1.14)*	WS wind speed max (m/s)	0.56 (0.28, 0.83)*
Age	0.02 (-0.01, 0.05)	Age	0.00 (-0.06, 0.06)
Annual household income>20k	-0.64 (-1.6, 0.31)	Annual household income>20k	-0.95 (-2.56, 0.66)
Education > high school	-0.35 (-1.23, 0.53)	Education > high school	-0.68 (-2.16, 0.82)
Body fat (%)	-0.06 (-0.13, 0.01)	Body fat (%)	-0.17 (-0.28, -0.05)*
Log (daily steps)	-0.08 (-0.29, 0.14)	Log (daily steps)	-0.48 (-1.1, 0.15)
Employed	0.13 (-0.86, 1.11)	Employed	0.93 (-0.73, 2.59)
Diabetic	-0.3 (-1.36, 0.76)	Diabetic	-0.72 (-2.51, 1.07)
Health condition in Fair[a]	-0.2 (-1.3, 0.9)	Health condition in Fair[a]	0.12 (-1.73, 1.98)
Health condition in Poor[a]	0.46 (-2.3, 3.22)	Health condition in Poor[a]	-0.05 (-4.68, 4.59)
Godin Inactivity	0.01 (-0.84, 0.86)	Godin Inactivity	-0.36 (-1.8, 1.08)
Intervention	-0.06 (-0.54, 0.42)	Intervention	-0.4 (-1.86, 1.06)
Rural residents	0.73 (-0.38, 1.85)	Rural residents	-0.88 (-3.12, 1.34)
Urban OutWor	2.28 (0.91, 3.65)*	Urban OutWor	7.02 (4.37, 9.68)*
Rain	0.23 (-0.09, 0.54)	Rain	-0.15 (-1.07, 0.77)
Intervention*Rural residents	-0.38 (-1.01, 0.25)	Intervention*Rural residents	0.42 (-1.56, 2.41)
Intervention*Urban OutWor	-1.81 (-2.57, -1.05)*	Intervention*Urban OutWor	-5.63 (-7.99, -3.27)*

Note: ‘*’ denotes a 95% confidence interval does not contain 0. Models were adjusted for participant age, annual household income level, education level, measured body fat (%), log(mean daily steps), employment, being diabetic, self-reported health condition, and Godin activity level. Urban resident group was the reference group.

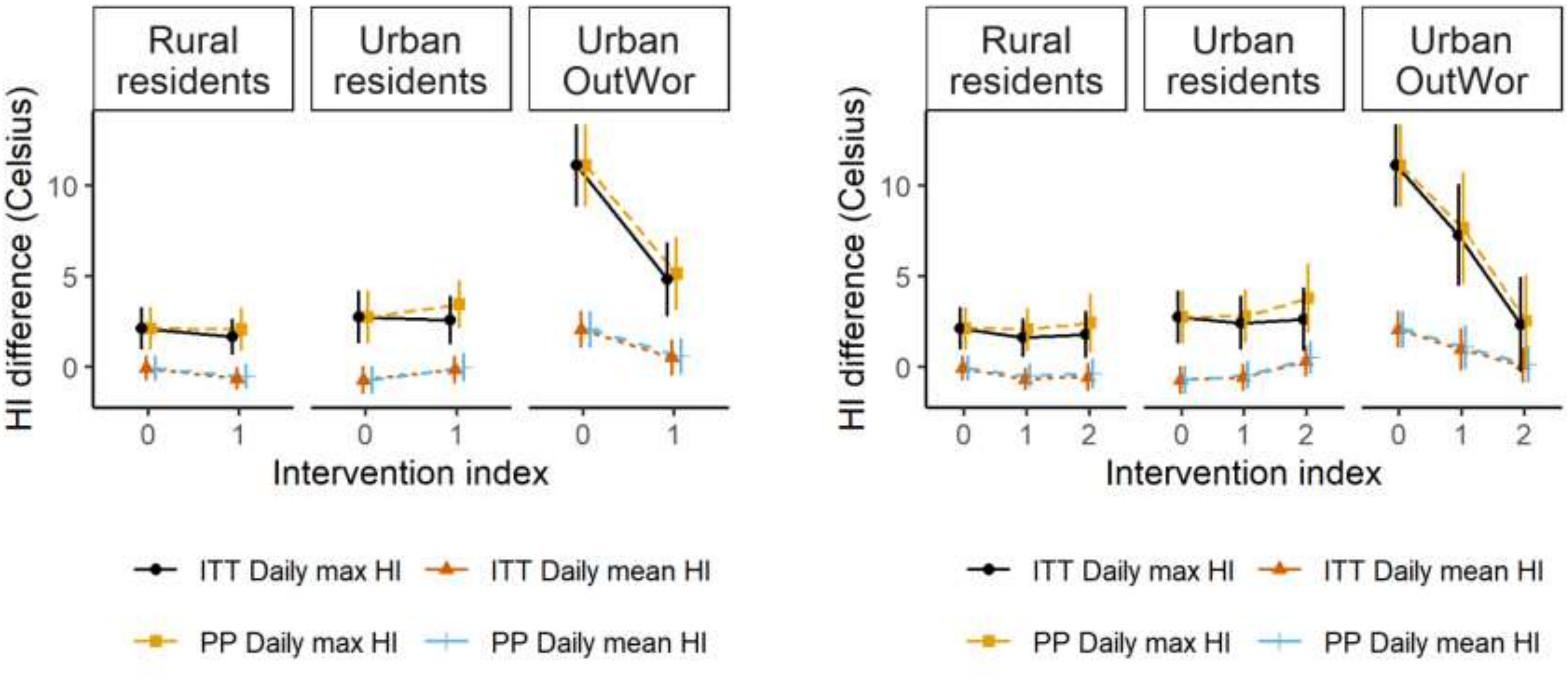

Supplemental File 8. Sensitivity analysis of intervention (or intervention &weekdays vs. intervention &weekend) effect on HI[individual] in ITT and PP. The population mean of HI difference(°C) between HI[individual] and HI[WS] on baseline days (intervention index = 0), intervention &weekdays (intervention index =1), and intervention &weekends (intervention index =2) in different population groups by using Intent-to-treat (ITT) and Per-Protocol (PP) dataset. The 95% confidence intervals are shown.

Supplemental File 8 (continued). Sensitivity analysis of intervention (or intervention &weekdays vs. intervention &weekend) effect on HI[individual] in ITT and PP. Results of linear mixed models describing the relation between the intervention (weekdays vs. weekends) and the daily *mean* heat index experienced by individuals in different population groups in ITT.

Group	All	Rural residents	Urban residents	Urban OutWor
Fixed effect	β 95%CI	β 95%CI	β 95%CI	β 95%CI
Intercept	28.96 (23.09, 34.81)	26.32 (17.22, 35.42)	35.3 (23.93, 46.66)	22.81 (7.34, 38.27)
HI[neighborhood] Mean (°C)	0.07 (-0.07, 0.22)	-0.04 (-0.26, 0.19)	-0.09 (-0.38, 0.21)	NA
HI[WS] Mean (°C)	NA	NA	NA	0.39 (-0.06, 0.83)
Wind speed Mean (m/s)	0.43 (-0.05, 0.91)	0.71 (-0.1, 1.52)	0.47 (-0.27, 1.21)	0.89 (-0.22, 2.01)
Age	0.02 (-0.02, 0.05)	0.01 (-0.04, 0.05)	0.04 (-0.01, 0.10)	0.03 (-0.08, 0.15)
Annual household income >20k	-0.75 (-1.67, 0.18)	-0.02 (-1.38, 1.35)	-0.96 (-2.86, 0.95)	-1.86 (-3.89, 0.18)
Education >high school	-0.36 (-1.24, 0.53)	-1.03 (-2.28, 0.23)	0.26 (-1.50, 2.02)	1.28 (-0.51, 3.08)
Body fat (%)	-0.06 (-0.13, 0.00)	0.05 (-0.06, 0.15)	-0.1 (-0.23, 0.03)	-0.13 (-0.25, -0.02)*
Log (daily steps)	-0.05 (-0.26, 0.16)	0.1 (-0.2, 0.39)	-0.41 (-0.77, -0.05)*	0.09 (-0.38, 0.56)
Employed	0.29 (-0.64, 1.21)	-0.41 (-1.72, 0.9)	1.01 (-0.63, 2.64)	NA
Diabetic	-0.17 (-1.21, 0.87)	0.1 (-1.23, 1.43)	-0.99 (-3.11, 1.13)	-2.6 (-6.13, 0.91)
Health condition in Fair[a]	-0.11 (-1.2, 0.99)	-0.89 (-2.41, 0.63)	1.22 (-0.71, 3.15)	-0.55 (-2.91, 1.81)
Health condition in Poor[a]	0.54 (-2.22, 3.3)	1.41 (-2.63, 5.46)	-2.03 (-7.44, 3.38)	-0.14 (-4.75, 4.47)
Godin inactivity	-0.03 (-0.89, 0.82)	0.00 (-1.27, 1.27)	0.38 (-1.18, 1.94)	-0.3 (-2.05, 1.45)
Intervention*weekday	-0.38 (-0.7, -0.07)*	-0.38 (-0.82, 0.06)	-0.21 (-0.74, 0.32)	-1.01 (-1.73, -0.29)*
Intervention*weekends	-0.98 (-1.36, -0.61)*	-0.64 (-1.12, -0.16)*	-0.66 (-1.49, 0.16)	-2.85 (-3.68, -2.01)*
WS Rain	0.09 (-0.24, 0.41)	0.23 (-0.18, 0.64)	-0.05 (-0.7, 0.61)	0.05 (-0.78, 0.87)

Note: ‘*’ indicates a 95% confidence interval does not contain 0. NA in HI[neighborhood] or HI[WS] daily mean indicates that HI[neighborhood] or HI[WS] daily mean was not included in the model selection based on AIC. Models for all participants did not include a group factor.

Supplemental File 8 (continued). Sensitivity analysis of intervention (or intervention &weekdays vs. intervention &weekend) effect on HI[individual] in ITT and PP. Results of linear mixed models describing the relation between the intervention (weekdays vs. weekends) and the daily *max* heat index experienced by individuals in different population groups in ITT.

Group	All	Rural residents	Urban residents	Urban OutWor
Fixed effect	β 95%CI	β 95%CI	β 95%CI	β 95%CI
Intercept	49.22 (37.34, 61.04)	36.39 (19.93, 52.86)	55.35 (33.26, 77.29)	10.81 (-39.98, 61.64)
HI[neighborhood] Max (°C)	0.04 (-0.18, 0.27)	0.04 (-0.26, 0.33)	0.00 (-0.45, 0.45)	NA
HI[WS] Max (°C)	NA	NA	NA	1.44 (0.10, 2.79)*
Wind speed Max (m/s)	0.49 (0.21, 0.76)*	0.25 (-0.11, 0.60)	1.01 (0.53, 1.5)*	0.36 (-0.51, 1.23)
Age	-0.01 (-0.07, 0.05)	-0.01 (-0.08, 0.07)	0.04 (-0.05, 0.13)	0.08 (-0.16, 0.32)
Annual household income >20k	-0.74 (-2.34, 0.86)	0.86 (-1.22, 2.93)	-4.02 (-7.03, -1.01)*	-2.71 (-7.00, 1.6)
Education >high school	-0.72 (-2.24, 0.80)	-0.84 (-2.74, 1.08)	-0.76 (-3.58, 2.06)	0.83 (-2.96, 4.68)
Body fat (%)	-0.21 (-0.33, -0.10)*	0.00 (-0.16, 0.16)	-0.18 (-0.39, 0.02)	-0.29 (-0.53, -0.06)*
Log (daily steps)	-0.37 (-1.00, 0.26)	-0.03 (-0.81, 0.76)	-1.37 (-2.46, -0.28)*	-0.55 (-2.24, 1.14)
Employed	1.73 (0.12, 3.34)*	-0.78 (-2.76, 1.22)	3.55 (1.00, 6.11)*	NA
Diabetic	-0.71 (-2.5, 1.09)	-0.17 (-2.19, 1.86)	-1.62 (-4.90, 1.67)	-4.47 (-11.94, 2.87)
Health condition in Fair[a]	0.43 (-1.46, 2.32)	-0.66 (-2.97, 1.66)	1.13 (-1.86, 4.11)	-1 (-5.97, 4.01)
Health condition in Poor[a]	0.65 (-4.07, 5.37)	-0.55 (-6.68, 5.58)	-11.15 (-19.52, -2.77)*	7.42 (-2.04, 16.9)
Godin inactivity	-0.49 (-1.96, 0.98)	-0.67 (-2.6, 1.26)	-0.83 (-3.32, 1.65)	0.16 (-3.52, 3.81)
Intervention*weekday	-0.74 (-1.71, 0.23)	-0.01 (-1.25, 1.23)	-0.77 (-2.50, 0.97)	-4.43 (-7.26, -1.61)*
Intervention*weekends	-2.22 (-3.24, -1.2)*	-0.49 (-1.75, 0.78)	-0.68 (-2.63, 1.28)	-8.77 (-11.6, -5.94)*
WS Rain	-0.09 (-1.02, 0.84)	0.92 (-0.09, 1.93)	-2.32 (-4.32, -0.34)*	-1.89 (-5.2, 1.42)

Note: '*' indicates a 95% confidence interval does not contain 0. NA in HI[neighborhood] or HI[WS] daily max indicates that HI[neighborhood] or HI[WS] daily max was not included in the model selection based on AIC. Models for all participants did not include a group factor.

Supplemental File 8 (continued). Sensitivity analysis of intervention (or intervention &weekdays vs. intervention &weekend) effect on HI[individual] in ITT and PP. Full results of linear mixed models describing the relation between the intervention and the daily *mean* heat index experienced by individuals during the intervention in different population groups in PP.

Group	All	Rural residents	Urban residents	Urban OutWor
Fixed effect	β 95%CI	β 95%CI	β 95%CI	β 95%CI
Intercept	24.28 (18.82, 29.74)	27.62 (18.61, 36.65)	30.25 (20.21, 40.27)	3.64 (-10.05, 17.31)
HI[WS] Mean (°C)	NA	NA	NA	0.94 (0.58, 1.31)*
HI[neighborhood] Mean (°C)	0.20 (0.07, 0.33)*	-0.09 (-0.32, 0.13)	0.05 (-0.18, 0.28)	NA
Wind speed Mean (m/s)	0.73 (0.23, 1.23)*	0.98 (0.19, 1.77)*	0.57 (-0.21, 1.36)	1.76 (0.59, 2.95)*
Age	0.01 (-0.02, 0.05)	0.00 (-0.05, 0.05)	0.04 (-0.01, 0.10)	0.04 (-0.08, 0.15)
Annual household income >20k	-0.8 (-1.74, 0.14)	-0.20 (-1.60, 1.21)	-1.04 (-2.95, 0.87)	-1.77 (-3.8, 0.26)
Education >high school	-0.37 (-1.27, 0.53)	-0.97 (-2.26, 0.32)	0.17 (-1.59, 1.93)	1.06 (-0.73, 2.85)
Body fat (%)	-0.06 (-0.13, 0.01)	0.05 (-0.05, 0.15)	-0.08 (-0.22, 0.05)	-0.12 (-0.23, -0.005)*
Log (daily steps)	-0.03 (-0.26, 0.2)	0.1 (-0.23, 0.42)	-0.37 (-0.76, 0.03)	0.28 (-0.23, 0.79)
Employed	0.27 (-0.67, 1.21)	-0.44 (-1.79, 0.91)	0.91 (-0.73, 2.55)	NA
Diabetic	-0.16 (-1.22, 0.9)	0.1 (-1.27, 1.47)	-1.03 (-3.15, 1.08)	-2.54 (-6.07, 0.96)
Health condition in Fair[a]	-0.02 (-1.14, 1.11)	-0.87 (-2.44, 0.71)	1.31 (-0.63, 3.24)	-0.61 (-2.99, 1.76)
Health condition in Poor[a]	0.71 (-2.1, 3.51)	1.89 (-2.31, 6.07)	-2.20 (-7.59, 3.18)	0.03 (-4.56, 4.62)
Godin inactivity	-0.04 (-0.91, 0.82)	0.00 (-1.31, 1.30)	0.38 (-1.19, 1.93)	-0.51 (-2.26, 1.26)
Intervention	-0.49 (-0.79, -0.20)*	-0.30 (-0.71, 0.11)	-0.18 (-0.73, 0.37)	-1.67 (-2.32, -1.01)*
WS Rain	0.27 (-0.07, 0.62)	0.35 (-0.06, 0.76)	0.07 (-0.63, 0.76)	0.15 (-0.74, 1.03)

Note: '*' indicates a 95% confidence interval does not contain 0. NA in HI[neighborhood] or HI[WS] daily mean indicates that HI[neighborhood] or HI[WS] daily mean was not included in the model selection based on AIC. Models for all participants did not include a group factor.

Supplemental File 8 (continued). Sensitivity analysis of intervention (or intervention &weekdays vs. intervention &weekend) effect on HI[individual] in ITT and PP. Full results of linear mixed models describing the relation between the intervention and the daily *max* heat index experienced by individuals during the intervention in different population groups in PP.

Group	All	Rural residents	Urban residents	Urban OutWor
Fixed effect	β 95%CI	β 95%CI	β 95%CI	β 95%CI
Intercept	45.65 (3.41, 57.78)	34.68 (17.3, 52.04)	53.82 (31.84, 75.62)	-3.54 (-52.6, 45.53)
HI[WS] Max (°C)	NA	NA	NA	1.82 (0.53, 3.13)*
HI[neighborhood] Max (°C)	0.07 (-0.16, 0.3)	0.05 (-0.26, 0.36)	-0.09 (-0.53, 0.35)	NA
Wind speed Max (m/s)	0.55 (0.26, 0.84)*	0.37 (-0.02, 0.75)	1.00 (0.48, 1.51)*	0.58 (-0.33, 1.48)
Age	-0.01 (-0.07, 0.05)	-0.02 (-0.09, 0.06)	0.03 (-0.06, 0.11)	0.09 (-0.15, 0.33)
Annual household income >20k	-0.62 (-2.26, 1.01)	0.75 (-1.44, 2.91)	-4.1 (-6.99, -1.19)*	-2.31 (-6.57, 1.95)
Education >high school	-0.85 (-2.40, 0.71)	-0.81 (-2.82, 1.22)	-0.76 (-3.45, 1.91)	0.53 (-3.21, 4.29)
Body fat (%)	-0.18 (-0.30, -0.07)*	0.01 (-0.15, 0.18)	-0.11 (-0.31, 0.09)	-0.29 (-0.53, -0.05)*
Log (daily steps)	-0.22 (-0.91, 0.47)	0.03 (-0.86, 0.92)	-1.06 (-2.2, 0.09)	-0.48 (-2.22, 1.26)
Employed	1.45 (-0.19, 3.10)	-0.99 (-3.07, 1.10)	2.73 (0.27, 5.2)*	NA
Diabetic	-0.94 (-2.79, 0.90)	-0.34 (-2.46, 1.81)	-2.01 (-5.13, 1.13)	-4.23 (-11.61, 3.00)
Health condition in Fair[a]	0.84 (-1.12, 2.81)	-0.51 (-2.98, 1.99)	1.66 (-1.2, 4.53)	0.01 (-5.09, 5.07)
Health condition in Poor[a]	0.93 (-3.92, 5.78)	0.57 (-6.06, 7.17)	-10.92 (-18.78, -3.03)*	7.94 (-1.30, 17.20)
Godin inactivity	-0.58 (-2.08, 0.91)	-0.69 (-2.72, 1.35)	-0.39 (-2.77, 1.97)	-0.52 (-4.22, 3.12)
Intervention	-0.99 (-1.90, -0.08)*	0.35 (-0.85, 1.55)	-0.22 (-1.89, 1.45)	-6.48 (-8.95, -4.01)*
WS Rain	-0.04 (-1.05, 0.96)	1.16 (0.08, 2.25)*	-2.61 (-4.63, -0.61)*	-3.19 (-6.38, 0.01)

Note: '*' indicates a 95% confidence interval does not contain 0. NA in HI[neighborhood] or HI[WS] daily max indicates that HI[neighborhood] or HI[WS] daily max was not included in the model selection based on AIC. Models for all participants did not include a group factor.

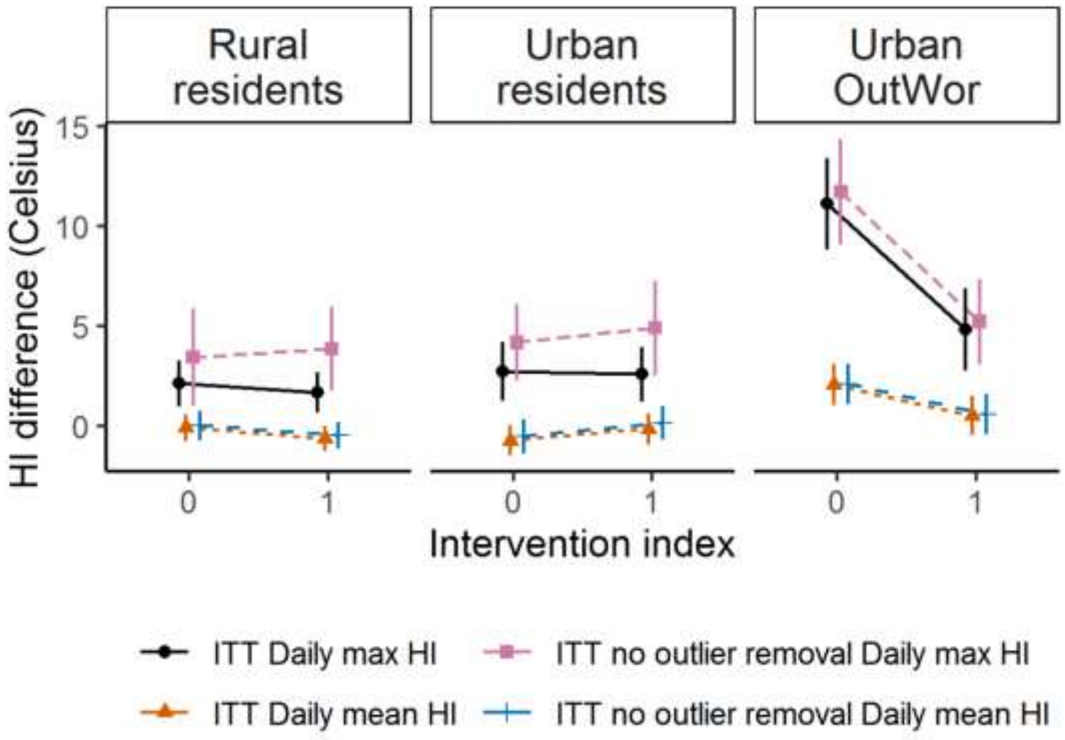

Supplemental File 9. The effect of ITT outlier removal on daily mean and max HI difference(°C) between HI[individual] and HI[WS]. The population mean or max of the daily mean HI difference(°C) between HI[individual] and HI[WS] on baseline days (intervention index = 0) and intervention days (intervention index =1) in different population groups in ITT and ITT-no outlier removal datasets. The 95% confidence intervals are shown.

Supplemental File 9 (continued). Full results of linear mixed models describing the relation between the intervention and the daily *mean or max* heat index experienced by individuals during the intervention across all participants in ITT-no outlier removal dataset.

Models	Model 1	Models	Model 2
Dependent variable	Daily mean HI[individual]	Dependent variable	Daily max HI[individual]
Fixed effect	β 95%CI	Fixed effect	β 95%CI
Intercept	24.29 (18.63, 29.95)	Intercept	52.72 (34.91, 70.38)
HI[WS] Mean (°C)	NA	HI[WS] Max (°C)	NA
HI[neighborhood] Mean (°C)	0.22 (0.08, 0.35)*	HI[neighborhood] Max (°C)	0.06 (-0.27, 0.40)
Wind speed Mean (m/s)	0.68 (0.17, 1.19)*	Wind speed Max (m/s)	0.30 (-0.12, 0.72)
Age	0.02 (-0.02, 0.05)	Age	0.01 (-0.08, 0.09)
Annual household income >20k	-0.81 (-1.76, 0.15)	Annual household income >20k	-1.7 (-4.07, 0.67)
Education >high school	-0.52 (-1.43, 0.39)	Education >high school	-1.56 (-3.79, 0.69)
Body fat (%)	-0.07 (-0.14, -0.001)*	Body fat (%)	-0.25 (-0.42, -0.08)*
Log (daily steps)	-0.03 (-0.26, 0.21)	Log (daily steps)	-0.58 (-1.55, 0.40)
Employed	0.47 (-0.49, 1.43)	Employed	3.19 (0.81, 5.57)*
Diabetic	-0.15 (-1.23, 0.93)	Diabetic	-0.43 (-3.09, 2.23)
Health condition in Faira	-0.58 (-1.72, 0.56)	Health condition in Faira	-0.81 (-3.61, 2.00)
Health condition in Poora	0.30 (-2.55, 3.14)	Health condition in Poora	-1.08 (-8.04, 5.88)
Godin inactivity	0.09 (-0.79, 0.97)	Godin inactivity	0.35 (-1.81, 2.52)
Intervention	-0.51 (-0.83, -0.19)*	Intervention	-0.58 (-1.93, 0.76)
WS Rain	0.22 (-0.13, 0.57)	WS Rain	-0.15 (-1.60, 1.29)

Note: ‘*’ indicates a 95% confidence interval does not contain 0. NA in HI[WS] daily mean indicates that HI[WS] daily mean was not included in the model selection based on AIC. Models for all participants did not include a group factor.

Supplemental File 10. Body measurement change ratios (%) of participants (N=177) including extreme body measurement change ratios.

Body measurement change ratio(%)[a]	Mean (95%CI)	Participant N	Obese level[b]	β 95%CI	Participant N
Body fat	-0.22 (-1.22, 0.79)	172	Normal	-0.92 (-3.56, 1.71)	9
			Overweight	-0.51 (-2.20, 1.18)	29
			Obese	-0.1 (-1.34, 1.13)	134
Body water	0.40 (-0.25, 1.05)	172	Normal	0.47 (-1.20, 2.15)	9
			Overweight	0.24 (-0.88, 1.35)	29
			Obese	0.43 (-0.36, 1.22)	134
Muscle mass	-1.32 (-1.99, -0.66)*	172	Normal	-0.42 (-2.39, 1.55)	9
			Overweight	-1.21 (-2.48, 0.07)	29
			Obese	-1.41 (-2.2, -0.61)*	134

Note: '*' denotes a 95% confidence interval does not contain 0.

[a] $Body\ measurement\ change\ ratio = \frac{body\ measurement_{after}\ - body\ measurement_{before}}{body\ measurement_{before}} \times 100\%$

[b]Obese level: Normal = BMI <25, Overweight = BMI <30 and ≥25, Obese = BMI ≥30.

This is the end of Supplemental Files.
